# Thalamic and Cerebellar Regional Involvement across the ALS–FTD Spectrum and the Effect of *C9orf72*

**DOI:** 10.3390/brainsci12030336

**Published:** 2022-03-01

**Authors:** Martina Bocchetta, Emily G. Todd, Nga Yan Tse, Emma M. Devenney, Sicong Tu, Jashelle Caga, John R. Hodges, Glenda M. Halliday, Muireann Irish, Olivier Piguet, Matthew C. Kiernan, Jonathan D. Rohrer, Rebekah M. Ahmed

**Affiliations:** 1 Dementia Research Centre, Department of Neurodegenerative Disease, UCL Queen Square Institute of Neurology, University College London, London WC1N 3BG, UK; emily.todd.18@ucl.ac.uk (E.G.T.); j.rohrer@ucl.ac.uk (J.D.R.); 2 Brain & Mind Centre, The University of Sydney, Sydney, NSW 2050, Australia; nga.tse@sydney.edu.au (N.Y.T.); emma.devenney@sydney.edu.au (E.M.D.); sicong.tu@sydney.edu.au (S.T.); jashelle.caga@sydney.edu.au (J.C.); john.hodges@sydney.edu.au (J.R.H.); glenda.halliday@sydney.edu.au (G.M.H.); matthew.kiernan@sydney.edu.au (M.C.K.); 3 School of Psychology, The University of Sydney, Sydney, NSW 2050, Australia; muireann.irish@sydney.edu.au (M.I.); olivier.piguet@sydney.edu.au (O.P.); 4 Memory and Cognition Clinic, Institute of Clinical Neurosciences, Royal Prince Alfred Hospital, Sydney, NSW 2050, Australia

**Keywords:** frontotemporal dementia, amyotrophic lateral sclerosis, magnetic resonance imaging

## Abstract

Amyotrophic lateral sclerosis (ALS) and frontotemporal dementia (FTD) are part of the same disease spectrum. While thalamic–cerebellar degeneration has been observed in *C9orf72* expansion carriers, the exact subregions involved across the clinical phenotypes of the ALS–FTD spectrum remain unclear. Using MRIs from 58 bvFTD, 41 ALS–FTD and 52 ALS patients compared to 57 controls, we aimed to delineate thalamic and cerebellar subregional changes across the ALS–FTD spectrum and to contrast these profiles between cases with and without *C9orf72* expansions. Thalamic involvement was evident across all ALS–FTD clinical phenotypes, with the laterodorsal nucleus commonly affected across all groups (values below the 2.5th control percentile). The mediodorsal nucleus was disproportionately affected in bvFTD and ALS–FTD but not in ALS. Cerebellar changes were only observed in bvFTD and ALS–FTD predominantly in the superior–posterior region. Comparison of genetic versus sporadic cases revealed significantly lower volumes exclusively in the pulvinar in *C9orf72* expansion carriers compared to non-carriers, irrespective of clinical syndrome. Overall, bvFTD showed significant correlations between thalamic subregions, level of cognitive dysfunction and severity of behavioural symptoms. Notably, strong associations were evident between mediodorsal nucleus atrophy and severity of behavioural changes in *C9orf72*-bvFTD (*r* = −0.9, *p* < 0.0005). Our findings reveal distinct thalamic and cerebellar atrophy profiles across the ALS–FTD spectrum, with differential impacts on behaviour and cognition, and point to a unique contribution of *C9orf72* expansions in the clinical profiles of these patients.

## 1. Introduction

Amyotrophic lateral sclerosis (ALS) and frontotemporal dementia (FTD) are often conceptualised as lying on a disease spectrum, with motor changes at one end and cognitive–behavioural changes at the other [1]. Accordingly, the syndrome of ALS–FTD lies somewhere in between these two phenotypes, with about 15% of ALS cases satisfying diagnostic criteria for bvFTD [2] and 10–15% of bvFTD patients eventually developing ALS [3,4,5]. These clinical syndromes share common neuropathological and genetic features, with the presence of 43 kDa TAR DNA-binding (TDP-43) protein deposition in the brain [6,7,8] and expansions in the chromosome 9 open reading frame 72 (*C9orf72*) gene [9,10]. Except for those patients who carry *C9orf72* expansions, who are likely to have TDP-43 pathology [11], it is currently not possible to identify the underlying pathology in life. Efforts to determine the neuroanatomical differences across this spectrum are crucial, enabling a better understanding of clinical phenotypes and disease trajectories.

Mounting evidence suggests that the thalamus and cerebellum play an important role in several relevant behavioural and clinical phenotypes in FTD and ALS, including neuropsychiatric and behavioural changes [12,13,14] and altered eating behaviour [15]. Both structures undergo significant atrophy in FTD, especially in *C9orf72* expansion carriers [16,17], and in ALS patients with and without *C9orf72* expansions [17,18,19,20,21]. Both structures, however, are highly heterogeneous, comprising anatomically distinct subregions, with discrete connectivity and functional profiles [16]. To our knowledge, so far, no study has explored how subregions of the thalamus and cerebellum are differentially impacted across the ALS–FTD spectrum or studied the impact of the *C9orf72* expansion on these structures.

The current study aimed to delineate the pattern of involvement of the subregions of the thalamus and the cerebellum and in a well-defined cohort of patients spanning the ALS–FTD spectrum. We further sought to determine how subregional atrophy of the thalamus and cerebellum relates to clinical and cognitive markers of disease. We hypothesised that distinct profiles of subregional involvement would be evident across each clinical phenotype and that the presence of *C9orf72* expansions would modify the pattern of atrophy and clinical correlates relative to sporadic cases.

## 2. Materials and Methods

In total, 99 individuals diagnosed with bvFTD (*n* = 58, mean (standard deviation) age 62.2 (8.3) years; 66% male) or ALS–FTD (*n* = 41, mean (standard deviation) age 65.0 (8.2) years; 76% male) were recruited from the FRONTIER clinic, the multidisciplinary clinical research group specialising in FTD and related younger-onset dementias. Additionally, 52 individuals diagnosed with ALS (mean (standard deviation) age 60.8 (10.8) years; 81% male) were recruited from the multidisciplinary FOREFRONT ALS and FTD clinic, specialising in the diagnosis and management of motor neurodegenerative syndromes. Both clinics are based at the Brain and Mind Centre at the University of Sydney, Australia. Patients were included in each diagnostic group based on their phenotypic presentation rather than on their family history or genetic status. Diagnostic assessment consisted of a medical and neurological examination, comprehensive neuropsychological assessment, clinical interviews, and a structural brain MRI. Functional assessment in the ALS and ALS–FTD patients at initial presentation was carried out using the revised version of the ALS functional rating scale (ALSFRS-R [22,23]).

Diagnosis was determined by multidisciplinary consensus by a senior neurologist, clinical neurophysiologist, and clinical neuropsychologist in accordance with current clinical diagnostic criteria [24,25,26]. ALS patients were classified as pure ALS (no cognitive changes) or ALS–FTD. Patients with ALS with cognitive or behavioural impairment who did not meet criteria for ALS–FTD were not included. All patients underwent screening for the *C9orf72* expansions and for ALS- or FTD-associated mutations in *MAPT*, *GRN* and *SOD1*. It was found that 31 patients carried an expansion in *C9orf72* (bvFTD = 17; ALS–FTD = 12; ALS = 2), 3 bvFTD patients carried a mutation in *GRN* and one in *MAPT*, while one patient with ALS carried a mutation in *SOD1* (Table 1). Fifty-seven cognitively normal participants matched for age and education were included as controls (mean (standard deviation) age at baseline 64.2 (10.9) years; 44% male). Inclusion criteria for controls included a score > 88/100 on the third edition of the Addenbrooke’s Cognitive Examination (ACE-III [27]), to ensure the absence of significant cognitive impairment. Exclusion criteria for all participants included the presence of another dementia syndrome and/or psychiatric disorders. This study was approved by the South-Eastern Sydney Local Health District and the University of New South Wales and University of Sydney ethics committees. All the participants or the person responsible for them provided written, informed consent in accordance with the Declaration of Helsinki.

### 2.1. Clinical Data

All cognitive and behavioural measures were completed within 3 months of MRI acquisition. Assessment included the ACE-III, for which a total score and subdomain scores were available (attention, memory, fluency, language and visuospatial skills), and the revised version of the Cambridge Behavioural Inventory (CBI-R [28]) to measure behavioural symptoms, with a total score and 10 subdomain scores (memory and orientation, everyday skills, self-care, abnormal behaviour, mood changes, odd beliefs, abnormal eating habits, sleep, stereotypic and motor behaviours and reduced motivation).

### 2.2. Image Acquisition

The bvFTD and ALS–FTD group as well as 40 controls underwent volumetric MRI on a 3T Philips Achieva scanner, and a further 10 controls in a 3T General Electric (GE) scanner (both equipped with a standard 8-channel head coil) to obtain high-resolution T1-weighted image series using the following parameters (FTD protocol): matrix 256 × 256, 200 slices, 1 mm^2^ in-plane resolution, slice thickness = 1 mm, echo time = 2.6 ms, repetition time = 5.8 ms, flip angle = 8°). The ALS group and a separate group of control participants (*n* = 7) were scanned on the 3T GE scanner. High-resolution T1-weighted image series were acquired using the following parameters (ALS protocol): matrix 256 × 256, 200 slices, 1 mm^2^ in-plane resolution, slice thickness = 0.5 mm, echo time = 2.6 ms, repetition time = 5.8 ms, flip angle = 8°.

### 2.3. Thalamic and Cerebellar Subregions

**Figure 1:**
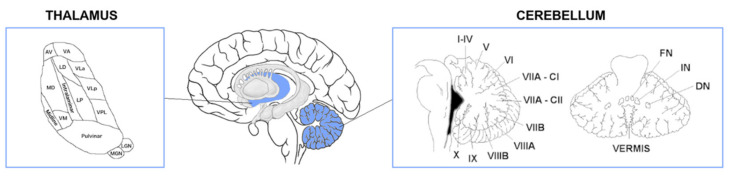
Regions of interest used in the analysis. Abbreviations. Thalamus: AV—anteroventral, VA—ventral anterior, LD—laterodorsal, VLa—ventral lateral anterior, MD—mediodorsal, LP—lateral posterior, VLp—ventral lateral posterior, VPL—ventral posterolateral, VM—ventromedial, LGN—lateral geniculate nucleus, MGN—medial geniculate nucleus. Cerebellum: VIIA-CI—lobule VIIA Crus I, VIIA-CII—lobule VIIA Crus II, FN—fastigial nucleus, IN—interposed nucleus, DN—dentate nucleus.

Volumetric T1-weighted MRI scans were bias-field corrected and whole-brain parcellated using the geodesic information flow (GIF) algorithm [29], which is based on atlas propagation and label fusion. The following cerebellar volumes were extracted: lobules I-IV, V, VI, VIIa-Crus I, VIIa-Crus II, VIIb, VIIIa, VIIIb, IX, X, vermis, dentate nucleus, interposed nucleus and fastigial nucleus [30,31]. Using GIF and a customised version of the FreeSurfer module [32] that accepts the GIF parcellations as inputs, we also calculated individual volumes for the following thalamic regions: anteroventral (AV), laterodorsal (LD), lateral posterior (LP), ventral anterior (VA), ventral lateral anterior (VLa), ventral lateral posterior (VLp), ventral posterolateral (VPL), ventromedial (VM), intralaminar, midline, mediodorsal (MD), lateral geniculate (LGN), medial geniculate (MGN) and pulvinar (Figure 1).

Left and right volumes were summed, and the total intracranial volume (TIV) was computed with SPM12 v6217 (Statistical Parametric Mapping, Wellcome Trust Centre for Neuroimaging, London, UK) running under MATLAB R2014b (Math Works, Natick, MA, USA) [33]. All segmentations were visually checked for quality.

To correct for different variables, and especially for the presence of different imaging acquisition protocols and scanners, we computed the w-scores of the brain volumes from controls, using the following formula: w-score = ((observed volume in patient) − (predicted patient volume))/(square root of the residual variance), where the predicted patient volume and the residual variance in controls were estimated from a linear regression model carried out on the volumes of the controls adjusting for the effect of age, sex, TIV, acquisition protocol and scanner type. In the control group, w-scores have a mean value of 0 and a standard deviation of 1; a w-score of −1.96 corresponds to the 2.5th percentile of the controls, −1.65 to the 5th percentile and −1.04 to the 15th percentile, respectively.

### 2.4. Statistical Analyses

Statistical analyses were performed in Stata v.14 (Stata Statistical Software: College Station, TX: StataCorp LP) and SPSS v.26 software (SPSS Inc., Chicago, IL, USA). First, we carried out bootstrapping with 1000 replicates to verify whether thalamic w-scores and cerebellar w-scores in each of the clinical groups (bvFTD, ALS–FTD, ALS) were significantly different from 0, indicating volumes below the mean average of controls. We also directly compared w-scores between the clinical groups. Next, for each of the clinical diagnoses (bvFTD, ALS–FTD, ALS), we compared sporadic and *C9orf72* expansion subgroups to controls. We also performed Bonferroni corrected *t*-tests to investigate differences in w-scores between sporadic versus *C9orf72* expansion carriers in the bvFTD group and sporadic versus *C9orf72* expansion carriers in the ALS–FTD group. *MAPT*, *GRN* and *SOD1* mutation carriers were excluded from all subgroup analyses.

Spearman’s rho correlation analyses were performed in each clinical group (bvFTD, ALS–FTD, ALS) and genetic subgroup (i.e., sporadic bvFTD, sporadic ALS–FTD, bvFTD *C9orf72* expansion carriers, ALS–FTD *C9orf72* expansion carriers) separately to test associations between thalamic and cerebellar w-scores and global cognitive function (ACE-III total score) and overall behavioural dysfunction (CBI-R total score). Statistical significance was set at a more conservative level of *p* < 0.01 to control for Type I error. Correlations were not performed in the ALS *C9orf72* subgroup, as only two patients were present.

## 3. Results

### 3.1. Characteristics of Study Participants

#### 3.1.1. Demographics

Demographic, clinical and genetic data are reported in Table 1 and were characteristic of the disease phenotypes. No significant group differences were found in education level or age across all groups (*p* = 0.303 and *p* = 0.267, *t*-test); however, sex distribution differed between the ALS and control groups, with more females present in the control group (*p* < 0.0005, chi-square test). There was no difference in sex among the clinical and genetic groups (*p* = 0.182 and *p* = 0.436, chi-square test). Similarly, groups did not differ in terms of age (*p* = 0.324 and *p* = 0.096, ANOVA) or education (*p* = 0.627 and *p* = 0.152, ANOVA). As expected, bvFTD had a longer disease duration compared to both ALS–FTD (*p* = 0.001) and ALS (*p* < 0.0005). No significant difference was present between the ALS–FTD and ALS groups on the ALSFRS-R score (*p* = 0.229) (Table 1). There was no significant difference in disease duration or ALSFRS-R score between the genetic and sporadic groups within each of the three clinical groups.

#### 3.1.2. Cognitive Function

BvFTD and ALS–FTD, but not ALS, showed lower overall cognitive function on the ACE-III total and subscales compared to controls (*p* ≤ 0.001). In contrast, the ALS group outperformed the other clinical groups across all ACE-III subdomain scores (*p* ≤ 0.01), while the ACE-III language subdomain was disproportionately affected in ALS–FTD compared to bvFTD (*p* = 0.008). These profiles are consistent with previous reports in the literature [34]. There was no significant difference in ACE-III total scores between the genetic and sporadic groups within each of the three clinical groups.

#### 3.1.3. Behavioural Changes

Compared to the ALS group, both bvFTD and ALS–FTD showed significantly greater behavioural disturbances (CBI-R total score: *p* < 0.001 and *p* = 0.012), spanning all CBI-R subscales, except for mood, sleep and everyday and self-care skills (Table 1). In addition, bvFTD showed significantly greater impairment than ALS–FTD in the overall behaviours (CBI-R total score: *p* = 0.004), particularly for abnormal behaviours, eating habits, sleep changes, reduced motivation, and mood changes. There was no significant difference in CBI-R total scores between the genetic and sporadic groups within each of the three clinical groups.

### 3.2. Imaging Findings

#### 3.2.1. Thalamic and Cerebellar Regional Atrophy Patterns Compared to Controls

**Figure 2:**
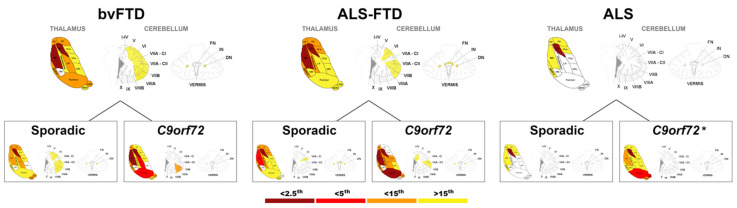
Pattern of neuroanatomical involvement in bvFTD, ALS–FTD and ALS groups, further divided by the sporadic form or presence of *C9orf72* expansions. The colour map indicates the percentile corresponding to the average w-score in each patient group, when this was statistically negative when compared to controls. * Only two ALS patients carried a *C9orf72* expansion, and the relative figure should be interpreted with caution. Abbreviations. Thalamus: AV—anteroventral, VA—ventral anterior, LD—laterodorsal, VLa—ventral lateral anterior, MD—mediodorsal, LP—lateral posterior, VLp—ventral lateral posterior, VPL—ventral posterolateral, VM—ventromedial, LGN—lateral geniculate nucleus, MGN—medial geniculate nucleus. Cerebellum: VIIA-CI—lobule VIIA Crus I, VIIA-CII—lobule VIIA Crus II, FN—fastigial nucleus, IN—interposed nucleus, DN—dentate nucleus.

When examining the thalamic volumes, bvFTD patients showed significantly lower volumes in all subregions of the thalamus (i.e., significantly negative w-scores) compared with controls, except for the VM (Table 2, Figure 2). In particular, LD and MD were below the 2.5th percentile of the control distribution, while the AV, LP, VA, midline, LGN and the pulvinar were below the 15th percentile (Table 2, Figure 2). Similarly, the ALS–FTD group also showed significantly lower volumes in all thalamic subregions, with values below the 2.5th percentile in the MD and LD, and below the 15th percentile in the AV, VA, VLa, intralaminar and midline. Lastly, the ALS group showed comparatively circumscribed atrophy with significantly lower volumes only observed in LD (below the 2.5th percentile), and in AV, VA, VLa, midline and MD (Table 2, Figure 2).

On examination of cerebellar volumes, bvFTD patients showed significantly lower cerebellar volumes than controls in lobule V, VI, VIIa-Crus I, VIIa-Crus II, VIIb, VIIIa and in the dentate nuclei (Table 2, Figure 2). In the ALS–FTD group, significantly lower volumes were observed in all the deep nuclei of the cerebellum and in the lobule VI, VIIa-Crus II and VIIb. In contrast, no cerebellar regional atrophy was evident in the ALS group relative to the controls (Table 2, Figure 2).

#### 3.2.2. Thalamic and Cerebellar Regional Atrophy Patterns across Clinical Diagnosis

Table 3 shows direct comparisons of thalamic and cerebellar subregional volumes between the clinical diagnoses. For the thalamic subregions, bvFTD patients showed significantly lower w-scores (more atrophy) than both ALS and ALS–FTD in the LP (*p* = 0.041), and significantly lower w-scores than ALS in all other thalamic regions, except for AV, LD, VA and VLa. Similarly, ALS–FTD showed significantly lower w-scores than ALS in all thalamic regions, except for LD and VA.

For the cerebellar subregions, both bvFTD and ALS–FTD showed significantly lower w-scores than ALS in all cerebellar regions, except for lobule VI, X and the interposed and fastigial nuclei (Table 3). No significant differences were found between the bvFTD and ALS–FTD groups.

#### 3.2.3. Effect of Genetic Status on Thalamic Regional Atrophy

##### bvFTD

Compared with controls, both sporadic bvFTD and bvFTD *C9orf72* carriers showed significant atrophy in all thalamic regions (except for VPL and VM), with the *C9orf72* group showing more extreme values. In particular, both genetic and sporadic groups showed values for LD below the 2.5th percentile and below the 15th percentile for AV and LGN (Table 2, Figure 2). In addition, bvFTD *C9orf72* carriers showed significantly lower values than controls for the following regions: MD (<2.5th percentile), LP and pulvinar (<5th percentile) and midline values (<15th percentile). In the sporadic bvFTD group, significantly lower values than controls were also found in the following regions: MD (<5th percentile) and VA, LP and MGN (<15th percentile) (Table 2, Figure 2). 

Finally, direct comparisons between the genetic and sporadic bvFTD subgroups revealed significantly lower w-scores in the pulvinar and midline nuclei in *C9orf72* expansion carriers compared to sporadic bvFTD patients (Table 3).

##### ALS–FTD

Compared with controls, sporadic ALS–FTD showed significantly lower w-scores in all thalamic subregions (except for LGN and VM) and most notably in the LD (<2.5th percentile) and MD (<5th percentile), while the AV, VA, VLa, intralaminar and midline showed significantly lower w-scores above the 15th percentile of controls (Table 2, Figure 2).

Genetic ALS–FTD cases showed significantly lower values than controls for the following regions: LD, MD and pulvinar (<2.5th percentile); AV, LP, VLp, intralaminar, midline and LGN (<15th percentile) and VPL (>15th percentile) (Table 2, Figure 2).

The direct comparison of the genetic and sporadic ALS–FTD subgroups revealed significantly lower w-scores in the pulvinar, MD and LGN in *C9orf72* expansion carriers compared to sporadic groups (Table 3).

##### ALS

Finally, sporadic ALS patients showed significantly lower values relative to controls in the LD (<2.5th percentile) and in the AV, VA, VLa, midline and MD (>15th percentile) (Table 2, Figure 2). The two *C9orf72* expansion carriers with a diagnosis of ALS showed all w-score values lower than controls, most pronounced for the LD (<2.5th percentile), pulvinar and LGN (<5th percentile) and MD and MGN (<15th percentile) (Table 2, Figure 2).

#### 3.2.4. Effect of Genetic Status on Cerebellar Regional Volumes

##### bvFTD

Compared to controls, *C9orf72* expansion carriers with a diagnosis of bvFTD showed significantly lower volumes in lobules VIIb and VIIIa (<15th percentile) (Figure 2). Sporadic bvFTD cases showed significantly lower w-scores than controls in lobule V, VI, VIIa-Crus II, VIIb and VIIIa and in the dentate nuclei (Table 2, Figure 2). No significant differences were detected between genetic and sporadic patients with bvFTD (Table 3).

##### ALS–FTD

Considering the ALS–FTD group, significantly lower values were found in the sporadic group in lobule VIIa-Crus I and the deep nuclei, while *C9orf72* expansion carriers showed significantly lower values in lobule I–IV, VIIa-Crus II and dentate nuclei compared to controls (Table 2, Figure 2). When directly compared to sporadic cases, *C9orf72* expansion carriers showed significantly lower w-scores in lobule I–IV (Table 3).

##### ALS

Finally, no significant differences were found between sporadic ALS and controls for any of the cerebellar regions (Table 2, Figure 2).

### 3.3. Correlations between Thalamic Volumes and Behaviour and Cognition

Table S1 shows the correlations between clinical and cognitive total scores within each clinical diagnostic group. In bvFTD, significant negative associations were found between overall behavioural disturbances (CBI-R total score) and the VA, VLa, VLp, VPL and MD, denoting an association between lower thalamic volumes and increased behavioural changes (see Table S2 for CBI-R subdomain correlations). No significant associations were found between thalamic subregions and overall behavioural changes in ALS–FTD or ALS. 

**Figure 3:**
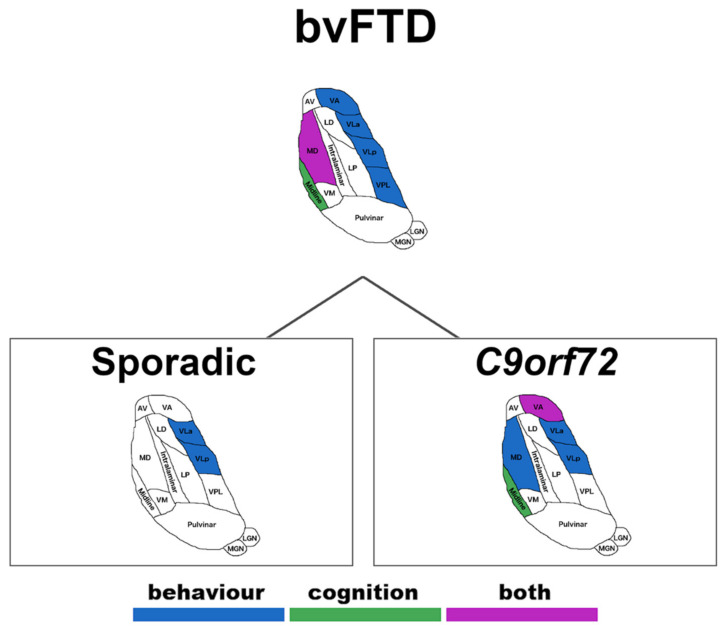
Pattern of neuroanatomical significant associations between thalamic regions and behavioural and cognitive performance in bvFTD, further divided by sporadic form or presence of *C9orf72* expansions. Abbreviations. Thalamus: AV—anteroventral, VA—ventral anterior, LD—laterodorsal, VLa—ventral lateral anterior, MD—mediodorsal, LP—lateral posterior, VLp—ventral lateral posterior, VPL—ventral posterolateral, VM—ventromedial, LGN—lateral geniculate nucleus, MGN—medial geniculate nucleus.

With regard to cognition, lower cognitive performance (ACE-III total score) in bvFTD was associated with lower volumes in the midline and MD (Table S1, see Table S2 for ACE-III subscale correlations). No significant associations were found between thalamic subregions and overall cognitive performance in ALS–FTD or ALS. The significant associations are shown in Figure 3.

#### Sporadic versus Genetic Correlations

In sporadic bvFTD, significant negative correlations (rho between −0.4 and −0.5) were found between VLa and VLp and overall behavioural change on the CBI-R total score (Table S1). In contrast, cognitive performance was not associated with any thalamic region in sporadic bvFTD (Tables S1 and S2).

*C9orf72* expansion carriers with a diagnosis of bvFTD showed a robust correlation of −0.9 (*p* < 0.0005) between MD and total CBI-R total score (Table S1). In addition, overall cognitive performance was positively associated with the midline and VA (Tables S1 and S2). No association was found between thalamic w-scores and the total scores of CBI-R nor ACE-III in genetic or sporadic groups for ALS–FTD and ALS (Table S1). The significant associations are shown in Figure 3.

### 3.4. Correlations between Cerebellar Volumes and Behaviour and Cognition

No association was found between cerebellar w-scores and the total scores of CBI-R or ACE-III in all groups (Table S1), and no association was found when looking at subdomains in bvFTD (sporadic and whole group) or in ALS (Table S2).

When examining the cerebellar subregions, bvFTD *C9orf72* expansion carriers showed associations between motivation and lobule VI, eating habits and dentate nuclei, visuospatial and lobule VIIIb, whilst in ALS–FTD *C9orf72* expansion carriers, lobule X was negatively associated with motivation on the CBI-R (Table S2).

## 4. Discussion

The current study employed automated segmentation methods on T1-weighted MR images to examine the pattern of involvement of thalamic and cerebellar regions in a well-characterised cohort of patients diagnosed with bvFTD, ALS–FTD and ALS, with and without *C9orf72* expansions. The study found significant thalamic subregions and cerebellar involvement across the ALS–FTD spectrum, with significant associations with cognitive and behavioural change, reinforcing the pivotal role that the thalamus and cerebellum play in the neurodegenerative process across the ALS–FTD spectrum.

Considering first the thalamus, we found extensive thalamic atrophy across the FTD–ALS spectrum in line with previous studies [19,20,35,36]. Our fine-grained parcellation method, however, uncovered profiles of atrophy to specific subregions across the FTD–ALS spectrum in vivo. Given the central role of the thalamus as a hub in several large-scale functional brain networks [37], it is not surprising that this structure should be widely affected in dementia. The region commonly affected across all clinical groups was the LD, with values below the 2.5th percentile of controls. The LD is a limbic region involved in learning, emotional experience and motivation [38], and damage to this region may, in part, account for many of the behavioural and socioemotional changes characteristic of these disorders [39,40]. Interestingly, however, we did not find significant associations between LD volume and cognitive or behavioural changes in any of the clinical syndromes on the screening measures used, which could be explained by the effect of a limbic component not necessarily captured by the scales used in this study or by the fact that LD volumes might not be a measure of disease severity, as measured by the scales. Both bvFTD and ALS–FTD additionally exhibited abnormal MD values, as previously reported [41] and in line with a pathological study that demonstrated neuronal loss in bvFTD [42]. This seems to suggest that the “FTD component” targets this thalamic region, which in turn would compromise structural and functional connectivity with the prefrontal, temporal and limbic areas, necessary for executive control and socioemotional regulation [38,41]. Moreover, both bvFTD and ALS–FTD showed overall more thalamic atrophy than pure ALS, both ventro-anteriorly (despite not significantly different) and posteriorly. In a previous study on the same cohort [34], we reported more subcortical involvement in the bvFTD and ALS–FTD groups than in ALS, which instead showed more cortical atrophy, specifically in the frontoparietal, insular, motor cortices and brainstem. These regions are connected to the thalamic areas that we have now found to be affected in ALS [41]. The areas particularly abnormal in bvFTD and ALS–FTD are instead connected to the limbic areas, including subcortical structures and prefrontal regions, as previously reported [34,43].

Overall, our findings are largely in keeping with previous post-mortem studies demonstrating TDP-43 pathology mainly in the anterior, laterodorsal, mediodorsal and dorsomedial nuclei of the thalamus, with the lateral thalamus being less affected [44]. The current study has shown that changes in thalamic structures can be identified in vivo and could be indicative of involvement of the thalamus in an early phase of the disease process.

Considering next the impact of genetic status on thalamic volumes, we found that *C9orf72* expansion carriers exhibited more extensive thalamic atrophy, with more extreme negative w-scores relative to the sporadic groups. This was in line with another study looking at the extent of atrophy across the disease spectrum [43]. In our study, in particular, the pulvinar showed lower values in carriers compared to non-carriers in all clinical syndromes, confirming the specific involvement of this region in *C9orf72*-related neurodegenerative diseases [39,41,45]. Neuropathological studies [46,47] have found the accumulation of TDP-43 and dipeptide repeat proteins in the pulvinar, which may give rise to the affective and psychotic symptoms often seen in *C9orf72* expansion carriers [13,48,49]. *C9orf72* expansion carriers with ALS–FTD compared to those with bvFTD showed particularly abnormal values in the VA, VLa, VLp and LP regions, which are connected to motor and somatosensory cortical areas, as has been found previously [50]. 

Cerebellar atrophy is well established across the ALS–FTD spectrum [51,52]. Here, we found that both bvFTD and ALS–FTD were associated with abnormal volumes in the superior–posterior cerebellum. This subregion of the cerebellum is connected via the ventro-lateral and ventro-anterior thalamus to the prefrontal cortex and is typically involved in cognitive processing (executive functions, language, attention). In addition, this region is connected to the frontal cortex to regulate motor and premotor functions [53,54]. In contrast with previous studies [43,51,52], we did not find any significant changes in cerebellar subregions in the ALS group. It may be that parcellating the cerebellum into smaller subregions reduces our capacity to detect significant differences in this group. In parallel, differences in clinical severity, scanner type and imaging analysis could also have a bearing on the results.

As was seen for the thalamus, more abnormal cerebellar values were evident in genetic compared to sporadic bvFTD and ALS–FTD, mainly in the dentate nucleus and the superior–posterior cerebellum. In addition, ALS–FTD showed a statistically significant difference between carriers and non-carriers in lobule I–IV, a region typically involved in sensorimotor control [54]. Overall, the presence of *C9orf72* expansions is typically associated with greater thalamic and cerebellar atrophy across the ALS–FTD spectrum [19,45,55,56,57], leading to the suggestion that, despite not being unique to the genetic cases, abnormalities in these structures, most notably the thalamus [58], could be a signature of *C9orf72* expansions [57].

Independent of genotype, our correlation analyses revealed significant associations between the thalamic midline and MD subregions and overall cognitive function, resonating with perspectives that propose a fundamental role for the thalamus in flexible forms of cognition [59]. Our correlation analyses further revealed striking associations between the MD and level of behavioural changes exclusively in the *C9orf72* bvFTD group, similarly to what was reported for the whole thalamus by Spinelli and colleagues [43]. These results, together with the selective MD abnormality in bvFTD and ALS–FTD but not pure ALS, suggest that MD dysfunction could be central to the severity of behavioural and cognitive changes in FTD [41] and a potential imaging marker for disease progression. Surprisingly, we did not find any significant correlations between thalamic or cerebellar volumes and cognition and behaviour in ALS or ALS–FTD groups, nor was the cerebellum implicated in cognition or behaviour in bvFTD. Preliminary findings from our subscale correlations suggest that the dentate nuclei may be related to eating habits, lobule VI with motivation, and lobule VIIIb with visuospatial function in the genetic (but not sporadic) bvFTD cases. Whilst the dentate nuclei are the output from the cerebellum to the thalamus and hypothalamus and, therefore, could contribute to the development of eating behaviour disturbances [15], and whilst lobule VI has been previously associated with motivation in bvFTD [60], visuospatial tasks are usually associated with lobule VI, rather than with lobule VIIIb [58,60]. It will be important for future studies to build on our findings here in order to determine the precise nature of brain–behaviour associations across the ALS–FTD spectrum.

Finally, a number of abnormal behaviours measured by the CBI-R were correlated with the VA, VLp and VLa regions in the thalamus in both sporadic and genetic bvFTD, but with stronger correlations in the *C9orf72* expansion carriers, which also involved other regions such as the MD and intralaminar [15,48]. Among these, the VA is related to controlling complex behaviours and motivation, due to its connections with the limbic system. Interestingly, whilst the VLa and VLp were strongly correlated with behavioural changes, these two thalamic regions were not correlated with cognitive performance. These two regions are considered motor nuclei, given their connections to the motor and premotor cortex, and as such, they might play a role in goal-directed decision-making [41,61].

This study has some limitations. By computing w-scores, we controlled for a number of variables, including different scanners, acquisition protocols and sex, but this does not completely exclude the possibility of influences of these factors on the results. We excluded positive w-scores from interpretation, as these were likely due to technical issues (possibly the effect of different scanner type) rather than to a biological cause, indicating higher volumes in patients than in controls. Patients with cognitive changes and ALS who did not meet the criteria for ALS–FTD were excluded to provide a comparison of ALS–FTD versus pure ALS, future studies should examine changes in those with pure ALS versus those with cognitive and behavioural changes who do not meet the diagnosis of ALS–FTD. Moreover, we could not formally assess the role of *C9orf72* on pure ALS patients, as the sample size was too small. Future larger studies, including longitudinal visits, will help to clarify the nature of thalamic and cerebellar changes, the temporal ordering of such changes according to disease staging and how the spread of atrophy within the thalamic–cerebellar network relates to the emergence of cognitive, behavioural and motor symptoms across the ALS–FTD spectrum.

## 5. Conclusions

In summary, the current study provides a comprehensive characterisation of subregional changes in the thalamus and cerebellum across the ALS–FTD spectrum and offers new insights into how such structural changes might impact behaviour and cognition, particularly highlighting the involvement of different regions of these structures. By examining the regional pattern of thalamic and cerebellar involvement, we provide further evidence of the role that such regions play in the ALS–FTD spectrum, included the impact of *C9orf72* on brain–behaviour associations. These findings provide potential neural correlates for many of the significant behavioural changes that we see across the ALS–FTD spectrum that influence disease progression and survival. A better understanding of the involvement of the thalamus and cerebellum across the spectrum and the modifying role of genetics potentially allows not only for improved clinical phenotyping but also for a better understanding of disease pathogenesis, potentially leading to more targeted drug treatment development.

## Figures and Tables

**Table 1:** Demographic characteristics of study participants. Values denote means and (standard deviations). Abbreviations. ACE—Addenbrooke’s Cognitive Examination; ALS—amyotrophic lateral sclerosis; ALSFRS-R—ALS functional rating scale-revised; ALS–FTD—amyotrophic lateral sclerosis–frontotemporal dementia; bvFTD—behavioural-variant frontotemporal dementia; CBI-R—Cambridge Behavioural Inventory Revised version.

	Controls (*n* = 57)	bvFTD (*n* = 58)	ALS–FTD (*n* = 41)	ALS (*n* = 52)
Sex (M/F)	25/32	38/20	31/10	42/10
Age (years)	64.2 (10.9)	61.7 (8.2)	64.5 (8.3)	60.3 (10.7)
Education (years)	13.4 (2.6)	12.4 (3.1)	12.7 (3.2)	13.0 (2.6)
Disease duration (months)	--	61.0 (49.5)	32.9 (22.6)	28.0 (27.6)
*C9orf72* expansion carriers (number)	--	17	12	2
Carriers of mutations in other genes (number)	--	3 *GRN*, 1 *MAPT*	0	1 *SOD1*
ALSFRS-R score (/45)	--	--	41	42
ACE III total score (/100)	94.6 (3.4)	77.1 (15.7)	72.0 (14.4)	92.4 (5.5)
ACE—Attention (/18)	17.2 (0.9)	14.9 (2.9)	15.3 (2.6)	17.0 (1.4)
ACE—Memory (/26)	24.7 (1.6)	190 (5.3)	19.0 (5.2)	23.2 (4.3)
ACE—Fluency (/14)	12.1 (1.6)	6.9 (4.0)	5.0 (3.7)	11.3 (1.8)
ACE—Language (/26)	25.1 (0.9)	22.4 (4.3)	19.3 (4.6)	24.5 (2.0)
ACE—Visuospatial (/16)	15.5 (0.9)	14.0 (2.6)	13.7 (2.1)	15.5 (0.9)
CBI-R total score (/180)	--	65.2 (29.2)	44.2 (30.9)	25.5 (20.4)
Memory	--	43.3 (21.4)	34.7(23.8)	12.1 (12.9)
Everyday skills	--	31.0 (25.9)	20.9 (20.4)	18.0 (30.0)
Self-care skills	--	16.3 (25.7)	8.5 (16.4)	22.7 (31.3)
Mood changes	--	29.6 (22.2)	21.3 (20.4)	16.9 (15.7)
Odd beliefs	--	10.1 (17.2)	5.41 (12.61)	0.2 (1.4)
Abnormal behaviours	--	36.6 (23.4)	22.9 (22.4)	8.5 (12.2)
Eating habits	--	41.6 (30.5)	23.7(28.0)	8.6 (12.2)
Sleep	--	43.8 (25.0)	27.7(26.0)	34.1 (27.5)
Stereotypic and motor behaviours	--	41.5 (28.9)	34.6(30.7)	8.6 (12.7)
Reduced motivation	--	58.9 (30.1)	34.8(29.0)	13.6 (17.3)

**Table 2:** Thalamic and cerebellar regions for the clinical and genetic groups. Values denote mean and standard deviation (SD) for the w-scores. Italics indicates significantly negative w-scores. Abbreviations. Thalamus: AV—anteroventral, VA—ventral anterior, LD—laterodorsal, VLa—ventral lateral anterior, MD—mediodorsal, LP—lateral posterior, VLp—ventral lateral posterior, VPL—ventral posterolateral, VM—ventromedial, LGN—lateral geniculate nucleus, MGN—medial geniculate nucleus. Cerebellum: VIIA-CI—lobule VIIA Crus I, VIIA-CII—lobule VIIA Crus II, FN—fastigial nucleus, IN—interposed nucleus, DN—dentate nucleus. ALS—amyotrophic lateral sclerosis; ALS–FTD—amyotrophic lateral sclerosis–frontotemporal dementia; bvFTD—behavioural-variant frontotemporal dementia.

		Thalamus															Cerebellum													
**Clinical Diagnosis**		**AV**	**LD**	**LP**	**VA**	**VLa**	**VLp**	**VPL**	**VM**	**Intralaminar**	**Midline**	**MD**	**LGN**	**MGN**	**Pulvinar**	**Whole**	**I–IV**	**V**	**VI**	**VIIa-CI**	**VIIa-CII**	**VIIb**	**VIIIa**	**VIIIb**	**IX**	**X**	**DN**	**IN**	**FN**	**Whole**
**bvFTD (n = 58)**	**Mean**	*−1.14*	*−4.61*	*−1.53*	*−1.13*	*−0.85*	*−0.73*	*−0.29*	−0.05	*−0.72*	*−1.12*	*−1.96*	*−1.13*	*−1.03*	*−1.15*	*−1.42*	−0.29	*−0.34*	*−0.41*	*−0.38*	*−0.52*	*−0.73*	*−0.82*	−0.09	−0.18	0.18	*−0.32*	−0.10	0.02	*−0.60*
	**SD**	*1.06*	*0.46*	*1.11*	*1.06*	*0.94*	*1.00*	*1.07*	1.08	*0.85*	*1.19*	*1.18*	*1.05*	*0.82*	*0.93*	*0.77*	1.12	*1.00*	*1.17*	*1.07*	*1.10*	*1.32*	*1.16*	1.01	0.87	0.98	*1.12*	1.13	1.04	*1.02*
	***p*** **-value**	*0.001*	*0.001*	*0.001*	*0.001*	*0.001*	*0.001*	*0.044*	0.716	*0.001*	*0.001*	*0.001*	*0.001*	*0.001*	*0.001*	*0.001*	0.062	*0.016*	*0.008*	*0.008*	*0.001*	*0.001*	*0.001*	0.500	0.149	0.191	*0.043*	0.510	0.913	*0.001*
**ALS** **–** **FTD (n = 41)**	**Mean**	*−1.36*	*−4.74*	*−1.03*	*−1.41*	*−1.14*	*−1.02*	*−0.59*	*−0.34*	*−1.08*	*−1.54*	*−2.00*	*−0.58*	*−0.94*	*−0.91*	*−1.49*	−0.19	−0.17	*−0.41*	−0.29	*−0.57*	*−0.63*	−0.48	−0.16	−0.17	0.18	*−0.81*	*−0.47*	*−0.30*	*−0.53*
	**SD**	*0.76*	*0.38*	*0.91*	*1.14*	*1.01*	*0.97*	*1.00*	*0.93*	*0.75*	*0.81*	*1.08*	*1.40*	*1.27*	*1.27*	*1.03*	1.08	0.98	*0.98*	0.99	*1.08*	*1.71*	1.55	0.94	0.95	1.00	*1.07*	*0.97*	*0.80*	*1.09*
	***p*** **-value**	*0.001*	*0.001*	*0.001*	*0.001*	*0.001*	*0.001*	*0.001*	*0.040*	*0.001*	*0.001*	*0.001*	*0.015*	*0.001*	*0.001*	*0.001*	0.272	0.297	*0.005*	0.058	*0.003*	*0.028*	0.063	0.316	0.253	0.281	*0.001*	*0.003*	*0.027*	*0.007*
**ALS (n = 52)**	**Mean**	*−0.78*	*−4.66*	−0.05	*−0.99*	*−0.49*	−0.19	0.46	0.74	−0.02	*−0.43*	*−0.29*	1.30	−0.09	0.46	−0.01	0.74	1.00	0.10	0.35	0.05	1.14	1.34	0.34	0.58	0.40	0.09	−0.29	−0.30	0.69
	**SD**	*0.72*	*0.48*	0.89	*0.90*	*1.08*	1.05	1.15	1.03	0.71	*0.65*	*0.86*	1.44	1.08	1.18	0.88	0.82	0.97	1.34	1.23	1.00	1.39	1.22	0.95	1.15	0.89	1.42	1.31	1.32	1.17
	***p*** **-value**	*0.001*	*0.001*	0.706	*0.001*	*0.002*	0.178	0.004	0.001	0.821	*0.001*	*0.023*	0.001	0.537	0.014	0.930	0.001	0.001	0.604	0.053	0.735	0.001	0.001	0.011	0.002	0.003	0.617	0.117	0.114	0.001
**Genetic subgroups**		**AV**	**LD**	**LP**	**VA**	**VLa**	**VLp**	**VPL**	**VM**	**Intralaminar**	**Midline**	**MD**	**LGN**	**MGN**	**Pulvinar**	**Whole**	**I–IV**	**V**	**VI**	**VIIa-CI**	**VIIa-CII**	**VIIb**	**VIIIa**	**VIIIb**	**IX**	**X**	**DN**	**IN**	**FN**	**Whole**
**bvFTD sporadic (n = 41)**	**Mean**	*−1.04*	*−4.62*	*−1.43*	*−1.16*	*−0.77*	*−0.66*	−0.32	0.00	*−0.65*	*−0.93*	*−1.74*	*−1.08*	*−1.08*	*−0.95*	*−1.27*	−0.26	*−0.30*	*−0.50*	−0.30	*−0.36*	*−0.61*	*−0.74*	−0.03	−0.06	0.18	*−0.37*	−0.04	0.15	*−0.50*
	**SD**	*1.07*	*0.44*	*1.00*	*1.12*	*0.91*	*1.00*	1.14	1.18	*0.92*	*1.32*	*1.18*	*1.00*	*0.91*	*0.85*	*0.76*	1.15	*0.89*	*1.03*	0.97	*1.05*	*1.41*	*1.29*	1.09	0.81	0.86	*1.18*	1.12	1.04	*0.98*
	***p*** **-value**	*0.001*	*0.001*	*0.001*	*0.001*	*0.001*	*0.001*	0.072	0.990	*0.002*	*0.001*	*0.001*	*0.001*	*0.001*	*0.001*	*0.001*	0.164	*0.043*	*0.007*	0.058	*0.044*	*0.012*	*0.006*	0.848	0.621	0.187	*0.047*	0.809	0.387	*0.004*
**bvFTD *C9orf72* (n = 12)**	**Mean**	*−1.49*	*−4.62*	*−1.93*	*−0.79*	*−0.93*	*−0.88*	−0.17	−0.09	*−0.94*	*−1.54*	*−2.42*	*−1.28*	*−0.82*	*−1.74*	*−1.74*	−0.23	−0.55	−0.36	−0.59	−0.99	*−1.15*	*−1.15*	−0.17	−0.51	0.05	−0.20	−0.29	−0.44	*−0.92*
	**SD**	*0.97*	*0.53*	*1.23*	*0.95*	*1.04*	*1.06*	0.99	0.85	*0.65*	*0.64*	*1.10*	*1.01*	*0.55*	*0.89*	*0.81*	1.13	1.39	1.34	1.40	1.33	*1.24*	*0.78*	0.61	1.05	1.31	1.12	1.17	1.12	*1.15*
	***p*** **-value**	*0.003*	*0.001*	*0.001*	*0.015*	*0.012*	*0.014*	0.549	0.703	*0.003*	*0.001*	*0.002*	*0.006*	*0.004*	*0.001*	*0.001*	0.491	0.189	0.335	0.145	0.056	*0.011*	*0.002*	0.352	0.118	0.914	0.541	0.396	0.174	*0.012*
**ALS** **–** **FTD sporadic (n = 29)**	**Mean**	*−1.25*	*−4.74*	*−0.96*	*−1.38*	*−1.09*	*−0.92*	*−0.43*	−0.23	*−1.06*	*−1.61*	*−1.78*	−0.15	*−0.66*	*−0.45*	*−1.21*	0.04	−0.02	−0.43	*−0.43*	−0.41	−0.51	−0.43	−0.03	−0.23	0.14	*−0.81*	*−0.45*	*−0.41*	*−0.46*
	**SD**	*0.77*	*0.41*	*0.99*	*0.89*	*0.78*	*0.80*	*0.91*	0.85	*0.74*	*0.88*	*1.07*	1.06	*1.02*	*0.99*	*0.80*	1.12	0.98	1.09	*1.00*	1.09	1.79	1.42	1.00	1.03	1.05	*1.16*	*0.90*	*0.78*	*1.14*
	***p*** **-value**	*0.001*	*0.001*	*0.001*	*0.001*	*0.001*	*0.001*	*0.021*	0.170	*0.001*	*0.001*	*0.001*	0.486	*0.001*	*0.021*	*0.001*	0.864	0.894	0.052	*0.046*	0.054	0.144	0.121	0.858	0.230	0.506	*0.002*	*0.010*	*0.013*	*0.050*
**ALS** **–** **FTD *C9orf72* (n = 12)**	**Mean**	*−1.61*	*−4.74*	*−1.19*	−1.49	−1.28	*−1.24*	*−0.99*	−0.60	*−1.12*	*−1.39*	*−2.53*	*−1.63*	−1.60	*−2.02*	*−2.16*	*−0.75*	−0.51	−0.38	0.03	*−0.99*	−0.92	−0.59	−0.46	−0.05	0.27	*−0.83*	−0.52	−0.04	−0.71
	**SD**	*0.69*	*0.31*	*0.68*	1.64	1.45	*1.32*	*1.13*	1.11	*0.81*	*0.62*	*0.95*	*1.59*	1.60	*1.22*	*1.23*	*0.74*	0.95	0.67	0.92	*0.99*	1.53	1.91	0.74	0.72	0.91	*0.85*	1.15	0.81	1.01
	***p*** **-value**	*0.001*	*0.001*	*0.002*	0.051	0.063	*0.031*	*0.020*	0.087	*0.004*	*0.003*	*0.002*	*0.015*	0.069	*0.004*	*0.002*	*0.009*	0.107	0.073	0.896	*0.013*	0.079	0.345	0.067	0.829	0.344	*0.010*	0.199	0.864	0.056
**ALS sporadic (n = 49)**	**Mean**	*−0.75*	*−4.67*	−0.01	*−0.98*	*−0.46*	−0.16	0.51	0.78	−0.01	*−0.45*	*−0.26*	1.44	−0.03	0.57	0.06	0.76	1.04	0.16	0.31	0.03	1.13	1.34	0.31	0.61	0.39	0.10	−0.31	−0.32	0.70
	**SD**	*0.72*	*0.49*	0.91	*0.92*	*1.07*	1.05	1.15	1.02	0.72	*0.67*	*0.82*	1.33	1.05	1.09	0.82	0.83	0.97	1.36	1.25	1.03	1.41	1.25	0.94	1.17	0.91	1.45	1.34	1.36	1.20
	***p*** **-value**	*0.001*	*0.001*	0.938	*0.001*	*0.012*	0.317	0.008	0.001	0.961	*0.001*	*0.029*	0.001	0.827	0.002	0.630	0.001	0.001	0.404	0.095	0.834	0.001	0.001	0.019	0.001	0.005	0.618	0.117	0.108	0.002
**ALS *C9orf72* (n = 2)**	**Mean**	−1.01	*−4.42*	−0.57	−0.84	−0.71	−0.58	−0.76	−0.24	−0.28	−0.27	−1.42	−1.67	−1.57	−1.69	−1.43	0.38	0.56	−0.70	1.37	0.38	1.72	1.16	0.27	−0.10	0.38	−0.07	0.25	0.05	0.83
	**SD**	0.68	*0.44*	0.11	0.38	1.73	1.43	1.08	1.22	0.84	0.18	1.33	1.18	1.51	1.64	1.53	1.05	0.06	0.95	0.16	0.60	1.16	0.70	1.01	0.06	0.87	1.34	0.91	0.54	0.39

**Table 3:** Comparisons across diagnostic and genetic groups for the thalamic and cerebellar regions. Values denote p-values. Italics indicates significantly negative w-scores. Abbreviations. Thalamus: AV—anteroventral, VA—ventral anterior, LD—laterodorsal, VLa—ventral lateral anterior, MD—mediodorsal, LP—lateral posterior, VLp—ventral lateral posterior, VPL—ventral posterolateral, VM—ventromedial, LGN—lateral geniculate nucleus, MGN—medial geniculate nucleus. Cerebellum: VIIA-CI—lobule VIIA Crus I, VIIA-CII—lobule VIIA Crus II, FN—fastigial nucleus, IN—interposed nucleus, DN—dentate nucleus. ALS—amyotrophic lateral sclerosis; ALS–FTD—amyotrophic lateral sclerosis–frontotemporal dementia; bvFTD—behavioural-variant frontotemporal dementia.

		Thalamus															Cerebellum													
**Clinical Diagnosis**		**AV**	**LD**	**LP**	**VA**	**VLa**	**VLp**	**VPL**	**VM**	**Intralaminar**	**Midline**	**MD**	**LGN**	**MGN**	**Pulvinar**	**Whole**	**I–IV**	**V**	**VI**	**VIIa-CI**	**VIIa-CII**	**VIIb**	**VIIIa**	**VIIIb**	**IX**	**X**	**DN**	**IN**	**FN**	**Whole**
**bvFTD**	**ALS–FTD**	0.673	0.446	*0.041*	0.514	0.481	0.494	0.524	0.504	0.071	0.080	1.000	0.119	1.000	0.891	1.000	1.000	1.000	1.000	1.000	1.000	1.000	0.611	1.000	1.000	1.000	0.141	0.361	0.467	1.000
	**ALS**	0.099	1.000	*<0.0005*	1.000	0.186	*0.019*	*0.001*	*<0.0005*	*<0.0005*	*0.001*	*<0.0005*	*<0.0005*	*<0.0005*	*<0.0005*	*<0.0005*	*<0.0005*	*<0.0005*	0.075	*0.002*	*0.018*	*<0.0005*	*<0.0005*	0.069	*<0.0005*	0.697	0.242	1.000	0.379	*<0.0005*
**ALS–FTD**	**ALS**	*0.006*	1.000	*<0.0005*	0.150	*0.007*	*<0.0005*	*<0.0005*	*<0.0005*	*<0.0005*	*<0.0005*	*<0.0005*	*<0.0005*	*<0.0005*	*<0.0005*	*<0.0005*	*<0.0005*	*<0.0005*	0.118	*0.020*	*0.017*	*<0.0005*	*<0.0005*	*0.047*	*0.001*	0.797	*0.001*	1.000	1.000	*<0.0005*
**Genetic vs. sporadic**		**AV**	**LD**	**LP**	**VA**	**VLa**	**VLp**	**VPL**	**VM**	**Intralaminar**	**Midline**	**MD**	**LGN**	**MGN**	**Pulvinar**	**Whole**	**I–IV**	**V**	**VI**	**VIIa-CI**	**VIIa-CII**	**VIIb**	**VIIIa**	**VIIIb**	**IX**	**X**	**DN**	**IN**	**FN**	**Whole**
**bvFTD**	***p*** **-value**	0.160	0.973	0.184	0.252	0.588	0.500	0.656	0.774	0.216	*0.023*	0.064	0.542	0.192	*0.009*	0.066	0.919	0.551	0.728	0.521	0.139	0.214	0.182	0.547	0.197	0.728	0.659	0.497	0.098	0.247
**ALS–FTD**	***p*** **-value**	0.157	0.968	0.427	0.838	0.696	0.469	0.136	0.303	0.830	0.368	*0.029*	*0.005*	0.070	*0.002*	*0.017*	*0.014*	0.130	0.879	0.154	0.103	0.451	0.806	0.141	0.533	0.645	0.947	0.860	0.194	0.478

## Data Availability

Data will be available on request from the authors until 2030.

## Supplementary Materials

The following supporting information can be downloaded at: https://www.mdpi.com/article/10.3390/brainsci12030336/s1, Table S1: Spearman’s correlations between w-scores and behavioural and cognitive total scores across the clinical and genetic groups. Table S2: Spearman’s correlations between w-scores and behavioural and cognitive subscores across the clinical and genetic groups.
