# Supplementary material for: Thalamic and Cerebellar Regional Involvement across the ALS–FTD Spectrum and the Effect of C9orf72
Source: Brain Sci. 2022 Mar 1;12(3):336. doi: 10.3390/brainsci12030336 (PMC8945983; doi:10.3390/brainsci12030336)
Supplement: Supplementary file 1 [file brainsci-12-00336-s001.zip › brainsci-1602926-supplementary.pdf]

Table S1. **Spearman's correlations between w-scores and behavioural and cognitive total scores across the clinical and genetic groups**. Bold indicates significantly correlation (p<0.01). *Abbreviations. Thalamus: AV anteroventral, VA ventral anterior, LD laterodorsal, VLa ventral lateral anterior, MD mediodorsal, LP lateral posterior, VLp ventral lateral posterior, VPL ventral posterolateral, VM ventromedial, LGN lateral geniculate nucleus, MGN medial geniculate nucleus. ACE Addenbrooke's Cognitive Examination; ALS amyotrophic lateral sclerosis; ALS-FTD amyotrophic lateral sclerosis-frontotemporal dementia; bvFTD behavioural-variant frontotemporal dementia; CBI-R Cambridge Behavioural Inventory Revised version.*

	**bvFTD (n=58)**		**CBI-R Total score**	**ACE III Total score**
**Thalamus**	**AV**	**rho**	-0.224	0.334
		**p-value**	0.091	0.010
	**LD**	**rho**	-0.018	0.072
		**p-value**	0.891	0.593
	**LP**	**rho**	-0.306	0.295
		**p-value**	0.019	0.024
	**VA**	**rho**	**-0.364**	0.313
		**p-value**	**0.005**	0.017
	**VLa**	**rho**	**-0.611**	0.254
		**p-value**	**<0.0005**	0.055
	**VLp**	**rho**	**-0.589**	0.226
		**p-value**	**<0.0005**	0.088
	**VPL**	**rho**	**-0.345**	-0.009
		**p-value**	**0.008**	0.948
	**VM**	**rho**	-0.317	0.021
		**p-value**	0.015	0.878
	**Intralaminar**	**rho**	-0.259	0.237
		**p-value**	0.05	0.074
	**Midline**	**rho**	-0.279	**0.369**
		**p-value**	0.034	**0.004**
	**MD**	**rho**	**-0.476**	**0.48**
		**p-value**	**<0.0005**	**<0.0005**
	**LGN**	**rho**	0.06	-0.056
		**p-value**	0.652	0.675
	**MGN**	**rho**	-0.094	-0.086
		**p-value**	0.482	0.523
	**Pulvinar**	**rho**	-0.081	0.031
		**p-value**	0.544	0.82
	**Whole thalamus**	**rho**	**-0.504**	**0.341**
		**p-value**	**<0.0005**	**0.009**
**Cerebellum**	**Lobule I-IV**	**rho**	0.036	-0.151
		**p-value**	0.788	0.259
	**Lobule V**	**rho**	-0.066	-0.093
		**p-value**	0.621	0.489
	**Lobule VI**	**rho**	-0.171	0.087
		**p-value**	0.2	0.517

	**Lobule VIIa-CrusI**	**rho**	-0.257	-0.093
		**p-value**	0.052	0.487
	**Lobule VIIa-CrusII**	**rho**	-0.152	-0.043
		**p-value**	0.254	0.749
	**Lobule VIIb**	**rho**	-0.087	-0.049
		**p-value**	0.516	0.716
	**Lobule VIIIa**	**rho**	-0.104	0.015
		**p-value**	0.437	0.91
	**Lobule VIIIb**	**rho**	-0.126	-0.013
		**p-value**	0.347	0.924
	**Lobule IX**	**rho**	0.047	-0.081
		**p-value**	0.724	0.548
	**Lobule X**	**rho**	-0.076	-0.12
		**p-value**	0.569	0.371
	**Dentate nuclei**	**rho**	-0.174	-0.147
		**p-value**	0.192	0.27
	**Interposed nuclei**	**rho**	-0.015	-0.03
		**p-value**	0.914	0.823
	**Fastigial nuclei**	**rho**	0.125	-0.027
		**p-value**	0.351	0.838
	**Whole cerebellum**	**rho**	-0.236	-0.081
		**p-value**	0.075	0.546
	**ALS-FTD (n=41)**		**CBI-R Total score**	**ACE III Total score**
**Thalamus**	**AV**	**rho**	-0.06	0.079
		**p-value**	0.723	0.63
	**LD**	**rho**	-0.07	-0.32
		**p-value**	0.68	0.044
	**LP**	**rho**	0.148	-0.283
		**p-value**	0.382	0.077
	**VA**	**rho**	-0.308	0.058
		**p-value**	0.063	0.721
	**VLa**	**rho**	-0.055	-0.234
		**p-value**	0.748	0.145
	**VLp**	**rho**	0.027	-0.264
		**p-value**	0.874	0.1
	**VPL**	**rho**	0.057	-0.165
		**p-value**	0.739	0.308
	**VM**	**rho**	0.091	-0.192
		**p-value**	0.592	0.236
	**Intralaminar**	**rho**	-0.008	-0.071
		**p-value**	0.962	0.663
	**Midline**	**rho**	-0.112	0.196
		**p-value**	0.509	0.226

	**MD**	**rho**	-0.245	0.185
		**p-value**	0.144	0.253
	**LGN**	**rho**	-0.011	-0.021
		**p-value**	0.947	0.897
	**MGN**	**rho**	-0.217	0.122
		**p-value**	0.198	0.453
	**Pulvinar**	**rho**	-0.044	-0.165
		**p-value**	0.795	0.309
	**Whole thalamus**	**rho**	-0.043	-0.073
		**p-value**	0.799	0.653
**Cerebellum**	**Lobule I-IV**	**rho**	-0.102	-0.053
		**p-value**	0.548	0.745
	**Lobule V**	**rho**	0.081	-0.066
		**p-value**	0.632	0.687
	**Lobule VI**	**rho**	0.14	-0.135
		**p-value**	0.408	0.405
	**Lobule VIIa-CrusI**	**rho**	0.125	-0.022
		**p-value**	0.46	0.893
	**Lobule VIIa-CrusII**	**rho**	0.005	-0.031
		**p-value**	0.976	0.849
	**Lobule VIIb**	**rho**	0.083	0.048
		**p-value**	0.624	0.768
	**Lobule VIIIa**	**rho**	-0.014	0.016
		**p-value**	0.936	0.92
	**Lobule VIIIb**	**rho**	-0.11	-0.001
		**p-value**	0.517	0.997
	**Lobule IX**	**rho**	0.038	-0.035
		**p-value**	0.823	0.829
	**Lobule X**	**rho**	0.136	-0.366
		**p-value**	0.423	0.02
	**Dentate nuclei**	**rho**	0.182	-0.184
		**p-value**	0.281	0.256
	**Interposed nuclei**	**rho**	0.101	-0.156
		**p-value**	0.553	0.336
	**Fastigial nuclei**	**rho**	0.168	-0.106
		**p-value**	0.32	0.515
	**Whole cerebellum**	**rho**	0.062	-0.053
		**p-value**	0.717	0.744
	**ALS (n=52)**		**CBI-R Total score**	**ACE III Total score**
**Thalamus**	**AV**	**rho**	-0.105	0.271
		**p-value**	0.587	0.122
	**LD**	**rho**	-0.334	0.251
		**p-value**	0.077	0.153

	**LP**	**rho**	-0.018	0.073
		**p-value**	0.925	0.68
	**VA**	**rho**	0.062	-0.104
		**p-value**	0.75	0.558
	**VLa**	**rho**	0.069	-0.253
		**p-value**	0.723	0.149
	**VLp**	**rho**	0.127	-0.182
		**p-value**	0.511	0.302
	**VPL**	**rho**	0.195	-0.02
		**p-value**	0.311	0.91
	**VM**	**rho**	0.128	0.04
		**p-value**	0.508	0.824
	**Intralaminar**	**rho**	0.181	0.1
		**p-value**	0.348	0.573
	**Midline**	**rho**	0.21	0.057
		**p-value**	0.273	0.748
	**MD**	**rho**	0.2	-0.076
		**p-value**	0.299	0.669
	**LGN**	**rho**	0.01	0.403
		**p-value**	0.959	0.018
	**MGN**	**rho**	0.17	-0.077
		**p-value**	0.379	0.664
	**Pulvinar**	**rho**	0.095	0.35
		**p-value**	0.624	0.042
	**Whole thalamus**	**rho**	0.246	0.128
		**p-value**	0.199	0.471
**Cerebellum**	**Lobule I-IV**	**rho**	0.202	-0.106
		**p-value**	0.293	0.551
	**Lobule V**	**rho**	0.198	0.006
		**p-value**	0.303	0.975
	**Lobule VI**	**rho**	0.251	0.2
		**p-value**	0.189	0.256
	**Lobule VIIa-CrusI**	**rho**	0.039	0.055
		**p-value**	0.839	0.757
	**Lobule VIIa-CrusII**	**rho**	0.166	-0.075
		**p-value**	0.39	0.673
	**Lobule VIIb**	**rho**	0.251	-0.088
		**p-value**	0.189	0.622
	**Lobule VIIIa**	**rho**	0.164	0.168
		**p-value**	0.394	0.342
	**Lobule VIIIb**	**rho**	-0.014	0.227
		**p-value**	0.944	0.196
	**Lobule IX**	**rho**	0.196	-0.014

		**p-value**	0.307	0.938
	**Lobule X**	**rho**	0.035	0.053
		**p-value**	0.855	0.767
	**Dentate nuclei**	**rho**	0.402	0.068
		**p-value**	0.03	0.704
	**Interposed nuclei**	**rho**	-0.071	0.032
		**p-value**	0.714	0.859
	**Fastigial nuclei**	**rho**	-0.182	-0.394
		**p-value**	0.344	0.021
	**Whole cerebellum**	**rho**	0.114	0.077
		**p-value**	0.557	0.665
	**bvFTD sporadic (n=41)**		**CBI-R Total score**	**ACE III Total score**
**Thalamus**	**AV**	**rho**	-0.239	0.202
		**p-value**	0.133	0.205
	**LD**	**rho**	-0.16	0.028
		**p-value**	0.319	0.86
	**LP**	**rho**	-0.328	0.185
		**p-value**	0.037	0.247
	**VA**	**rho**	-0.239	0.278
		**p-value**	0.132	0.079
	**VLa**	**rho**	**-0.512**	0.208
		**p-value**	**0.001**	0.193
	**VLp**	**rho**	**-0.469**	0.159
		**p-value**	**0.002**	0.32
	**VPL**	**rho**	-0.317	-0.051
		**p-value**	0.043	0.75
	**VM**	**rho**	-0.26	-0.02
		**p-value**	0.101	0.899
	**Intralaminar**	**rho**	-0.213	0.178
		**p-value**	0.18	0.264
	**Midline**	**rho**	-0.24	0.239
		**p-value**	0.13	0.133
	**MD**	**rho**	-0.352	0.268
		**p-value**	0.024	0.091
	**LGN**	**rho**	0.037	0.126
		**p-value**	0.816	0.431
	**MGN**	**rho**	-0.152	-0.02
		**p-value**	0.342	0.901
	**Pulvinar**	**rho**	-0.069	-0.038
		**p-value**	0.668	0.812
	**Whole thalamus**	**rho**	**-0.426**	0.207
		**p-value**	**0.006**	0.195
**Cerebellum**	**Lobule I-IV**	**rho**	-0.001	-0.154

		**p-value**	0.995	0.336
	**Lobule V**	**rho**	-0.142	-0.072
		**p-value**	0.374	0.654
	**Lobule VI**	**rho**	-0.155	0.193
		**p-value**	0.333	0.226
	**Lobule VIIa-CrusI**	**rho**	-0.261	-0.091
		**p-value**	0.1	0.57
	**Lobule VIIa-CrusII**	**rho**	-0.22	-0.156
		**p-value**	0.167	0.329
	**Lobule VIIb**	**rho**	-0.211	-0.137
		**p-value**	0.186	0.393
	**Lobule VIIIa**	**rho**	-0.095	-0.033
		**p-value**	0.555	0.835
	**Lobule VIIIb**	**rho**	0.006	-0.092
		**p-value**	0.968	0.569
	**Lobule IX**	**rho**	0.152	-0.087
		**p-value**	0.343	0.591
	**Lobule X**	**rho**	-0.059	-0.1
		**p-value**	0.716	0.532
	**Dentate nuclei**	**rho**	-0.168	-0.246
		**p-value**	0.294	0.121
	**Interposed nuclei**	**rho**	0.004	-0.036
		**p-value**	0.981	0.824
	**Fastigial nuclei**	**rho**	0.112	0.146
		**p-value**	0.485	0.363
	**Whole cerebellum**	**rho**	-0.21	-0.11
		**p-value**	0.188	0.493
	**bvFTD *C9orf72* (n=12)**		**CBI-R Total score**	**ACE III Total score**
**Thalamus**	**AV**	**rho**	-0.51	0.622
		**p-value**	0.09	0.031
	**LD**	**rho**	0.035	0.315
		**p-value**	0.914	0.319
	**LP**	**rho**	-0.594	0.427
		**p-value**	0.042	0.167
	**VA**	**rho**	**-0.818**	**0.741**
		**p-value**	**0.001**	**0.006**
	**VLa**	**rho**	**-0.797**	0.51
		**p-value**	**0.002**	0.09
	**VLp**	**rho**	**-0.804**	0.524
		**p-value**	**0.002**	0.08
	**VPL**	**rho**	-0.406	0.147
		**p-value**	0.191	0.649
	**VM**	**rho**	-0.685	0.364

		**p-value**	0.014	0.245
	**Intralaminar**	**rho**	-0.559	0.378
		**p-value**	0.059	0.226
	**Midline**	**rho**	-0.601	**0.783**
		**p-value**	0.039	**0.003**
	**MD**	**rho**	**-0.93**	0.678
		**p-value**	**<0.0005**	0.015
	**LGN**	**rho**	-0.063	-0.203
		**p-value**	0.846	0.527
	**MGN**	**rho**	0.259	-0.559
		**p-value**	0.417	0.059
	**Pulvinar**	**rho**	-0.399	0.28
		**p-value**	0.199	0.379
	**Whole thalamus**	**rho**	**-0.944**	0.685
		**p-value**	**<0.0005**	0.014
**Cerebellum**	**Lobule I-IV**	**rho**	0.147	0.147
		**p-value**	0.649	0.649
	**Lobule V**	**rho**	0.14	-0.021
		**p-value**	0.665	0.948
	**Lobule VI**	**rho**	-0.308	0.329
		**p-value**	0.331	0.297
	**Lobule VIIa-CrusI**	**rho**	-0.077	-0.217
		**p-value**	0.812	0.499
	**Lobule VIIa-CrusII**	**rho**	0.168	0.168
		**p-value**	0.602	0.602
	**Lobule VIIb**	**rho**	0.133	0.322
		**p-value**	0.681	0.308
	**Lobule VIIIa**	**rho**	0	0.217
		**p-value**	1	0.499
	**Lobule VIIIb**	**rho**	-0.406	0.308
		**p-value**	0.191	0.331
	**Lobule IX**	**rho**	-0.252	0.042
		**p-value**	0.43	0.897
	**Lobule X**	**rho**	-0.021	-0.259
		**p-value**	0.948	0.417
	**Dentate nuclei**	**rho**	-0.469	0.503
		**p-value**	0.124	0.095
	**Interposed nuclei**	**rho**	-0.259	-0.014
		**p-value**	0.417	0.966
	**Fastigial nuclei**	**rho**	0.343	-0.51
		**p-value**	0.276	0.09
	**Whole cerebellum**	**rho**	-0.217	0.105
		**p-value**	0.499	0.746

	ALS-FTD sporadic (n=29)		CBI-R Total score	ACE III Total score
Thalamus	AV	rho	-0.073	0.012
		p-value	0.722	0.951
	LD	rho	0	-0.402
		p-value	1	0.034
	LP	rho	0.269	-0.337
		p-value	0.184	0.08
	VA	rho	-0.348	-0.001
		p-value	0.081	0.998
	VLa	rho	0.091	-0.312
		p-value	0.657	0.106
	VLp	rho	0.227	-0.363
		p-value	0.264	0.058
	VPL	rho	0.15	-0.133
		p-value	0.464	0.501
	VM	rho	0.161	-0.096
		p-value	0.432	0.626
	Intralaminar	rho	-0.024	-0.168
		p-value	0.906	0.393
	Midline	rho	-0.212	0.05
		p-value	0.298	0.8
	MD	rho	-0.142	0.135
		p-value	0.49	0.494
	LGN	rho	0.044	0.173
		p-value	0.831	0.38
	MGN	rho	-0.091	0.137
		p-value	0.658	0.487
	Pulvinar	rho	0.092	-0.199
		p-value	0.655	0.311
	Whole thalamus	rho	0.07	-0.166
		p-value	0.732	0.397
Cerebellum	Lobule I-IV	rho	0.038	0.012
		p-value	0.854	0.95
	Lobule V	rho	0.147	0.007
		p-value	0.473	0.972
	Lobule VI	rho	0.075	-0.156
		p-value	0.715	0.428
	Lobule VIIa-CrusI	rho	0.018	-0.088
		p-value	0.931	0.656
	Lobule VIIa-CrusII	rho	0.129	-0.013
		p-value	0.53	0.949
	Lobule VIIb	rho	0.068	0.179
		p-value	0.74	0.363

	**Lobule VIIIa**	**rho**	-0.119	0.21
		**p-value**	0.564	0.284
	**Lobule VIIIb**	**rho**	-0.18	0.13
		**p-value**	0.378	0.511
	**Lobule IX**	**rho**	-0.071	0.075
		**p-value**	0.73	0.705
	**Lobule X**	**rho**	0.236	-0.385
		**p-value**	0.245	0.043
	**Dentate nuclei**	**rho**	0.026	-0.305
		**p-value**	0.9	0.115
	**Interposed nuclei**	**rho**	0.024	-0.301
		**p-value**	0.908	0.12
	**Fastigial nuclei**	**rho**	0.169	-0.165
		**p-value**	0.409	0.401
	**Whole cerebellum**	**rho**	0.042	0.022
		**p-value**	0.837	0.912
	**ALS-FTD *C9orf72* (n=12)**		**CBI-R Total score**	**ACE III Total score**
**Thalamus**	**AV**	**rho**	-0.009	0.392
		**p-value**	0.979	0.208
	**LD**	**rho**	-0.336	0.189
		**p-value**	0.312	0.557
	**LP**	**rho**	-0.045	-0.196
		**p-value**	0.894	0.542
	**VA**	**rho**	-0.245	0.406
		**p-value**	0.467	0.191
	**VLa**	**rho**	-0.382	0.119
		**p-value**	0.247	0.713
	**VLp**	**rho**	-0.291	0.021
		**p-value**	0.385	0.948
	**VPL**	**rho**	-0.1	-0.336
		**p-value**	0.77	0.286
	**VM**	**rho**	0.036	-0.427
		**p-value**	0.915	0.167
	**Intralaminar**	**rho**	0.009	0.196
		**p-value**	0.979	0.542
	**Midline**	**rho**	0.173	0.615
		**p-value**	0.612	0.033
	**MD**	**rho**	-0.3	0.378
		**p-value**	0.37	0.226
	**LGN**	**rho**	0.018	-0.573
		**p-value**	0.958	0.051
	**MGN**	**rho**	-0.4	0.049
		**p-value**	0.223	0.88

	**Pulvinar**	**rho**	-0.155	-0.161
		**p-value**	0.65	0.618
	**Whole thalamus**	**rho**	-0.255	0.112
		**p-value**	0.45	0.729
**Cerebellum**	**Lobule I-IV**	**rho**	-0.455	-0.063
		**p-value**	0.16	0.846
	**Lobule V**	**rho**	-0.136	-0.161
		**p-value**	0.689	0.618
	**Lobule VI**	**rho**	0.255	-0.021
		**p-value**	0.45	0.948
	**Lobule VIIa-CrusI**	**rho**	0.364	0.098
		**p-value**	0.272	0.762
	**Lobule VIIa-CrusII**	**rho**	-0.064	-0.084
		**p-value**	0.853	0.795
	**Lobule VIIb**	**rho**	0.2	-0.273
		**p-value**	0.555	0.391
	**Lobule VIIIa**	**rho**	0.236	-0.538
		**p-value**	0.484	0.071
	**Lobule VIIIb**	**rho**	0.109	-0.441
		**p-value**	0.75	0.152
	**Lobule IX**	**rho**	0.382	-0.559
		**p-value**	0.247	0.059
	**Lobule X**	**rho**	-0.182	-0.301
		**p-value**	0.593	0.342
	**Dentate nuclei**	**rho**	0.664	0.147
		**p-value**	0.026	0.649
	**Interposed nuclei**	**rho**	0.373	0.308
		**p-value**	0.259	0.331
	**Fastigial nuclei**	**rho**	0.036	-0.091
		**p-value**	0.915	0.779
	**Whole cerebellum**	**rho**	0.1	-0.266
		**p-value**	0.77	0.404
	**ALS sporadic (n=49)**		**CBI-R Total score**	**ACE III Total score**
**Thalamus**	**AV**	**rho**	-0.117	0.231
		**p-value**	0.553	0.197
	**LD**	**rho**	-0.34	0.249
		**p-value**	0.077	0.162
	**LP**	**rho**	-0.035	0.037
		**p-value**	0.858	0.839
	**VA**	**rho**	0.073	-0.073
		**p-value**	0.711	0.687
	**VLa**	**rho**	0.086	-0.226
		**p-value**	0.663	0.206

	VLp	**rho**	0.143	-0.167
		**p-value**	0.467	0.353
	VPL	**rho**	0.195	-0.062
		**p-value**	0.321	0.732
	VM	**rho**	0.125	0.014
		**p-value**	0.528	0.937
	Intralaminar	**rho**	0.189	0.114
		**p-value**	0.336	0.526
	Midline	**rho**	0.2	0.063
		**p-value**	0.308	0.73
	MD	**rho**	0.198	-0.096
		**p-value**	0.312	0.594
	LGN	**rho**	0.007	0.352
		**p-value**	0.97	0.045
	MGN	**rho**	0.176	-0.105
		**p-value**	0.37	0.562
	Pulvinar	**rho**	0.096	0.313
		**p-value**	0.629	0.076
	Whole thalamus	**rho**	0.239	0.097
		**p-value**	0.22	0.59
Cerebellum	Lobule I-IV	**rho**	0.213	-0.085
		**p-value**	0.276	0.639
	Lobule V	**rho**	0.206	-0.023
		**p-value**	0.292	0.899
	Lobule VI	**rho**	0.245	0.202
		**p-value**	0.208	0.259
	Lobule VIIa-CrusI	**rho**	0.043	0.096
		**p-value**	0.827	0.595
	Lobule VIIa-CrusII	**rho**	0.184	-0.026
		**p-value**	0.349	0.887
	Lobule VIIb	**rho**	0.276	-0.042
		**p-value**	0.155	0.817
	Lobule VIIIa	**rho**	0.144	0.14
		**p-value**	0.464	0.438
	Lobule VIIIb	**rho**	-0.018	0.214
		**p-value**	0.928	0.231
	Lobule IX	**rho**	0.199	-0.027
		**p-value**	0.309	0.882
	Lobule X	**rho**	0.019	0.035
		**p-value**	0.925	0.849
	Dentate nuclei	**rho**	0.414	0.105
		**p-value**	0.028	0.562
		**rho**	-0.067	0.023

	**Interposed nuclei**	**p-value**	0.733	0.898
	**Fastigial nuclei**	**rho**	-0.169	-0.375
		**p-value**	0.39	0.032
	**Whole cerebellum**	**rho**	0.117	0.094
		**p-value**	0.552	0.603

Table S2. **Spearman's correlations between w-scores and behavioural and cognitive subscores across the clinical and genetic groups**. Bold indicates significantly correlation (p<0.01). *Abbreviations.* *Thalamus: AV anteroventral, VA ventral anterior, LD laterodorsal, VLa ventral lateral anterior, MD mediodorsal, LP lateral posterior, VLp ventral lateral posterior, VPL ventral posterolateral, VM ventromedial, LGN lateral geniculate nucleus, MGN medial geniculate nucleus. ACE Addenbrooke's Cognitive Examination; ALS amyotrophic lateral sclerosis; ALS-FTD amyotrophic lateral sclerosis-frontotemporal dementia; bvFTD behavioural-variant frontotemporal dementia; CBI-R Cambridge Behavioural Inventory Revised version.*

			**CBI-R**										**ACE III**				
	**bvFTD (n=58)**		**Memory**	**Everyday skills**	**Self-care skills**	**Mood changes**	**Odd beliefs**	**Abnormal behaviours**	**Eating habits**	**Sleep**	**Stereotypic and motor behaviours**	**Reduced motivation**	**Attention**	**Memory**	**Fluency**	**Language**	**Visuospatial**
**Thalamus**	**AV**	**rho**	-0.083	**-0.347**	**-0.405**	0.09	-0.204	0.014	-0.303	-0.158	-0.023	-0.178	0.039	0.178	**0.465**	**0.369**	0.29
		**p-value**	0.535	**0.008**	**0.002**	0.503	0.125	0.915	0.021	0.235	0.862	0.182	0.771	0.182	**<0.0005**	**0.004**	0.027
	**LD**	**rho**	-0.082	-0.149	-0.215	0.227	0.138	0.07	-0.008	-0.284	0.046	-0.092	-0.062	0.026	0.207	0.139	0.121
		**p-value**	0.539	0.264	0.105	0.086	0.302	0.602	0.955	0.031	0.733	0.493	0.645	0.848	0.119	0.3	0.366
	**LP**	**rho**	-0.228	-0.29	-0.233	-0.032	**-0.382**	-0.094	-0.315	-0.137	-0.15	-0.215	0.122	0.147	**0.388**	0.286	0.169
		**p-value**	0.085	0.027	0.078	0.813	**0.003**	0.483	0.016	0.304	0.262	0.106	0.362	0.27	**0.003**	0.029	0.206
	**VA**	**rho**	-0.167	**-0.383**	**-0.337**	0.014	-0.127	-0.235	**-0.413**	-0.136	-0.202	-0.282	0.117	0.23	0.336	**0.341**	0.121
		**p-value**	0.211	**0.003**	**0.01**	0.92	0.341	0.075	**0.001**	0.307	0.127	0.032	0.383	0.082	0.01	**0.009**	0.364
	**VLa**	**rho**	-0.288	**-0.529**	**-0.401**	-0.256	-0.327	**-0.406**	**-0.525**	**-0.359**	-0.315	**-0.433**	0.197	0.068	0.328	0.271	0.13
		**p-value**	0.028	**<0.0005**	**0.002**	0.052	0.012	**0.002**	**<0.0005**	**0.006**	0.016	**0.001**	0.137	0.613	0.012	0.04	0.331
	**VLp**	**rho**	-0.299	**-0.507**	**-0.377**	-0.274	-0.302	**-0.394**	**-0.514**	-0.303	-0.329	**-0.418**	0.189	0.034	0.292	0.294	0.138
		**p-value**	0.022	**<0.0005**	**0.004**	0.037	0.021	**0.002**	**<0.0005**	0.021	0.012	**0.001**	0.156	0.801	0.026	0.025	0.302
	**VPL**	**rho**	-0.102	-0.212	-0.157	-0.187	-0.179	-0.317	-0.332	-0.279	-0.184	**-0.411**	0.072	-0.038	-0.001	0.146	-0.022
		**p-value**	0.447	0.111	0.24	0.16	0.18	0.015	0.011	0.034	0.167	**0.001**	0.593	0.777	0.993	0.275	0.869
	**VM**	**rho**	-0.233	-0.157	-0.071	-0.11	-0.25	-0.286	-0.289	-0.178	-0.145	-0.252	0.055	0.004	-0.009	0.131	-0.019
		**p-value**	0.079	0.239	0.598	0.411	0.058	0.03	0.028	0.182	0.277	0.057	0.683	0.978	0.945	0.327	0.889
	**Intralaminar**	**rho**	-0.267	-0.263	-0.208	-0.062	-0.261	-0.122	-0.197	-0.107	-0.155	-0.194	0.167	0.11	0.288	0.302	0.288
		**p-value**	0.043	0.046	0.117	0.642	0.048	0.362	0.138	0.425	0.246	0.144	0.21	0.412	0.029	0.021	0.028
	**Midline**	**rho**	-0.305	**-0.357**	**-0.337**	0.051	-0.206	-0.089	-0.24	-0.207	-0.126	-0.217	0.159	0.205	**0.478**	**0.49**	0.314
		**p-value**	0.02	**0.006**	**0.01**	0.703	0.12	0.508	0.069	0.119	0.346	0.103	0.234	0.122	**<0.0005**	**<0.0005**	0.016
	**MD**	**rho**	-0.329	**-0.5**	**-0.41**	-0.101	**-0.421**	-0.172	**-0.387**	-0.228	-0.138	**-0.435**	0.25	0.332	**0.565**	**0.477**	0.265

		**p-value**	0.012	**<0.0005**	**0.001**	0.452	**0.001**	0.196	**0.003**	0.085	0.303	**0.001**	0.058	0.011	**<0.0005**	**<0.0005**	0.044
	**LGN**	**rho**	-0.078	0.094	0.112	0.075	0.044	0.002	0.175	0.152	-0.081	0.072	0	0.227	-0.269	-0.112	-0.291
		**p-value**	0.561	0.483	0.401	0.578	0.745	0.985	0.188	0.255	0.546	0.594	0.999	0.087	0.041	0.404	0.027
	**MGN**	**rho**	0.044	-0.209	-0.072	-0.093	0.106	-0.133	-0.026	-0.295	-0.016	-0.18	-0.016	-0.117	-0.13	0.175	-0.094
		**p-value**	0.741	0.116	0.589	0.488	0.429	0.321	0.846	0.025	0.903	0.177	0.902	0.382	0.33	0.19	0.484
	**Pulvinar**	**rho**	-0.062	-0.224	-0.03	0.203	-0.097	-0.113	-0.011	0.001	-0.035	-0.001	0.016	0.071	-0.043	0.115	-0.009
		**p-value**	0.646	0.091	0.822	0.126	0.47	0.4	0.932	0.994	0.793	0.992	0.904	0.598	0.747	0.389	0.949
	**Whole thalamus**	**rho**	-0.322	**-0.557**	**-0.374**	-0.05	**-0.374**	-0.263	**-0.399**	-0.29	-0.217	**-0.396**	0.199	0.228	**0.369**	**0.426**	0.164
		**p-value**	0.014	**<0.0005**	**0.004**	0.709	**0.004**	0.046	**0.002**	0.027	0.103	**0.002**	0.135	0.086	**0.004**	**0.001**	0.22
**Cerebellum**	**Lobule I-IV**	**rho**	-0.034	-0.015	-0.045	0.063	0.195	-0.077	0.039	-0.19	-0.041	0.165	-0.043	-0.244	-0.221	0.067	0.006
		**p-value**	0.801	0.909	0.736	0.636	0.142	0.565	0.772	0.154	0.761	0.216	0.746	0.065	0.095	0.617	0.966
	**Lobule V**	**rho**	0.204	-0.068	-0.24	0.016	0.021	-0.08	-0.145	-0.269	-0.019	-0.183	-0.055	-0.183	-0.054	0.183	0.117
		**p-value**	0.124	0.611	0.07	0.907	0.876	0.55	0.277	0.041	0.887	0.17	0.683	0.168	0.685	0.17	0.381
	**Lobule VI**	**rho**	-0.075	0.039	-0.178	-0.129	0.044	-0.23	-0.183	-0.064	0.025	-0.248	0.23	-0.033	0.148	0.127	0.156
		**p-value**	0.573	0.773	0.18	0.335	0.744	0.083	0.17	0.634	0.852	0.061	0.083	0.807	0.267	0.343	0.242
	**Lobule VIIa-CrusI**	**rho**	0.026	-0.134	-0.146	-0.223	-0.141	-0.323	-0.165	-0.219	-0.234	-0.329	0.032	-0.249	-0.007	0.132	0.021
		**p-value**	0.847	0.315	0.273	0.092	0.292	0.013	0.215	0.099	0.077	0.012	0.814	0.059	0.959	0.325	0.876
	**Lobule VIIa-CrusII**	**rho**	-0.075	-0.236	-0.107	-0.008	-0.174	-0.171	-0.114	-0.269	0.03	-0.245	0.112	-0.163	0.041	0.093	0.15
		**p-value**	0.577	0.074	0.425	0.955	0.19	0.2	0.395	0.041	0.822	0.064	0.401	0.222	0.762	0.487	0.261
	**Lobule VIIb**	**rho**	-0.168	-0.089	-0.02	0.028	-0.077	-0.11	-0.071	-0.332	0.036	-0.06	0.101	-0.181	-0.008	0.164	0.088
		**p-value**	0.207	0.505	0.881	0.835	0.565	0.412	0.596	0.011	0.786	0.654	0.449	0.173	0.952	0.217	0.51
	**Lobule VIIIa**	**rho**	-0.18	-0.12	-0.063	0.034	0.029	-0.088	-0.098	-0.328	-0.017	-0.028	0.261	-0.108	-0.052	0.009	0.177
		**p-value**	0.175	0.369	0.636	0.803	0.827	0.513	0.466	0.012	0.899	0.837	0.048	0.418	0.7	0.944	0.185
	**Lobule VIIIb**	**rho**	-0.099	-0.28	-0.271	0.005	0.168	-0.047	-0.133	-0.209	-0.051	-0.001	0.058	-0.097	0.023	-0.061	0.099
		**p-value**	0.462	0.033	0.04	0.968	0.209	0.728	0.318	0.116	0.705	0.992	0.663	0.469	0.862	0.652	0.46
	**Lobule IX**	**rho**	0.013	-0.139	-0.07	-0.006	0.025	0.04	0.027	-0.048	0.077	0.155	0.085	-0.007	-0.149	-0.038	0.037
		**p-value**	0.922	0.298	0.603	0.961	0.849	0.766	0.839	0.72	0.568	0.244	0.525	0.958	0.263	0.775	0.785
	**Lobule X**	**rho**	-0.131	-0.075	-0.118	-0.045	0.075	-0.088	0.022	-0.186	0.05	0.01	-0.045	-0.126	-0.026	-0.053	0.087

		**p-value**	0.327	0.576	0.377	0.74	0.577	0.511	0.872	0.162	0.709	0.942	0.738	0.347	0.847	0.692	0.515
	**Dentate nuclei**	**rho**	-0.143	-0.156	-0.218	0.095	0.111	-0.066	-0.217	-0.142	-0.099	-0.159	-0.069	-0.067	-0.044	-0.057	-0.209
		**p-value**	0.285	0.241	0.1	0.477	0.409	0.62	0.102	0.288	0.459	0.233	0.606	0.618	0.741	0.672	0.115
	**Interposed nuclei**	**rho**	-0.027	0.051	-0.014	0.084	-0.154	-0.013	-0.036	0.03	-0.15	0.036	-0.043	0.014	0.056	-0.016	-0.119
		**p-value**	0.84	0.704	0.919	0.53	0.249	0.923	0.787	0.822	0.261	0.79	0.749	0.919	0.679	0.908	0.375
	**Fastigial nuclei**	**rho**	0.103	0.052	0.051	0.136	-0.083	0.157	0.193	-0.046	0.052	0.133	-0.062	0.03	-0.07	-0.105	-0.146
		**p-value**	0.44	0.699	0.706	0.309	0.534	0.238	0.147	0.731	0.7	0.321	0.645	0.823	0.602	0.434	0.275
	**Whole cerebellum**	**rho**	-0.078	-0.18	-0.278	-0.113	-0.079	-0.236	-0.22	-0.334	-0.065	-0.255	0.109	-0.263	0.039	0.143	0.114
		**p-value**	0.563	0.176	0.034	0.4	0.556	0.075	0.097	0.01	0.629	0.054	0.415	0.046	0.773	0.285	0.395
	**ALS-FTD (n=41)**		**Memory**	**Everyday skills**	**Self-care skills**	**Mood changes**	**Odd beliefs**	**Abnormal behaviours**	**Eating habits**	**Sleep**	**Stereotypic and motor behaviours**	**Reduced motivation**	**Attention**	**Memory**	**Fluency**	**Language**	**Visuospatial**
**Thalamus**	**AV**	**rho**	0.062	0.118	-0.203	0.154	-0.214	0.083	-0.136	0.198	0.048	0.117	0.174	0.026	0.09	-0.138	0.209
		**p-value**	0.709	0.476	0.214	0.349	0.192	0.615	0.411	0.228	0.772	0.478	0.296	0.876	0.591	0.41	0.208
	**LD**	**rho**	-0.06	0.14	0.049	-0.007	0.018	0.016	0.034	0.181	0.007	0.082	-0.302	-0.142	-0.274	-0.334	-0.321
		**p-value**	0.717	0.395	0.767	0.964	0.914	0.922	0.837	0.27	0.967	0.619	0.066	0.395	0.096	0.04	0.05
	**LP**	**rho**	0.273	0.367	0.01	0.218	-0.193	0.171	0.035	0.209	0.14	0.129	-0.069	-0.17	-0.357	-0.317	0.006
		**p-value**	0.092	0.022	0.95	0.183	0.239	0.299	0.835	0.202	0.394	0.433	0.68	0.307	0.028	0.053	0.974
	**VA**	**rho**	-0.14	0.022	-0.027	0.005	-0.277	-0.014	-0.306	0.026	-0.227	-0.091	0.104	0.047	0.051	-0.163	0.183
		**p-value**	0.396	0.896	0.872	0.976	0.087	0.934	0.058	0.874	0.164	0.582	0.534	0.781	0.762	0.328	0.271
	**VLa**	**rho**	0.11	0.286	0.05	0.233	-0.185	0.086	-0.138	0.077	-0.185	0.081	-0.066	-0.056	-0.324	-0.344	0.064
		**p-value**	0.506	0.077	0.762	0.154	0.259	0.604	0.401	0.64	0.259	0.626	0.693	0.738	0.047	0.035	0.702
	**VLp**	**rho**	0.207	0.362	0.019	0.296	-0.061	0.054	-0.094	0.152	-0.136	0.168	-0.011	-0.057	-0.39	-0.408	0.051
		**p-value**	0.206	0.024	0.908	0.067	0.712	0.743	0.568	0.357	0.41	0.306	0.946	0.733	0.016	0.011	0.761
	**VPL**	**rho**	0.196	0.126	-0.074	0.143	0.046	-0.089	-0.109	-0.035	-0.222	0.125	0.005	-0.044	-0.294	-0.18	0.051
		**p-value**	0.231	0.445	0.653	0.387	0.782	0.589	0.51	0.834	0.174	0.448	0.977	0.795	0.073	0.279	0.761
	**VM**	**rho**	0.22	0.088	-0.045	0.181	0.026	0.025	-0.082	-0.063	-0.194	0.031	-0.057	-0.065	-0.261	-0.219	0.085
		**p-value**	0.178	0.592	0.786	0.269	0.877	0.881	0.618	0.701	0.237	0.852	0.735	0.696	0.114	0.186	0.611
	**Intralaminar**	**rho**	0.013	0.195	-0.004	0.137	-0.127	0.141	-0.097	-0.073	-0.258	-0.019	0.01	0.034	0.043	-0.284	0.093

		**p-value**	0.935	0.234	0.983	0.404	0.442	0.393	0.556	0.659	0.113	0.91	0.952	0.837	0.8	0.084	0.577
	**Midline**	**rho**	-0.02	-0.091	0.024	0.041	-0.144	0.148	-0.214	-0.184	-0.119	-0.012	0.219	0.028	0.361	-0.126	0.286
		**p-value**	0.903	0.581	0.884	0.804	0.38	0.368	0.191	0.262	0.472	0.942	0.187	0.867	0.026	0.451	0.082
	**MD**	**rho**	-0.067	-0.048	-0.281	0.076	-0.267	-0.054	-0.368	-0.143	-0.126	-0.101	0.261	0.168	0.167	-0.033	0.279
		**p-value**	0.686	0.77	0.084	0.647	0.1	0.742	0.021	0.386	0.445	0.54	0.113	0.314	0.317	0.842	0.09
	**LGN**	**rho**	0.027	-0.059	-0.281	0.104	-0.222	-0.221	-0.225	-0.151	-0.064	-0.207	0.026	-0.045	-0.133	0.061	-0.04
		**p-value**	0.869	0.721	0.083	0.528	0.175	0.176	0.169	0.358	0.698	0.205	0.877	0.789	0.427	0.716	0.812
	**MGN**	**rho**	0.032	0.046	-0.11	-0.095	-0.155	-0.279	-0.385	-0.093	-0.343	-0.073	0.262	0.164	-0.01	-0.015	0.13
		**p-value**	0.847	0.783	0.504	0.564	0.345	0.086	0.015	0.573	0.032	0.657	0.113	0.325	0.95	0.927	0.438
	**Pulvinar**	**rho**	0.086	0.202	-0.09	0.15	-0.32	0.026	-0.178	-0.012	-0.024	0.009	-0.011	0.059	-0.339	-0.233	0.089
		**p-value**	0.602	0.217	0.585	0.362	0.047	0.874	0.278	0.941	0.883	0.956	0.948	0.726	0.037	0.159	0.596
	**Whole thalamus**	**rho**	0.141	0.227	-0.078	0.231	-0.259	0.095	-0.239	0.028	-0.12	0.066	0.123	0.084	-0.207	-0.239	0.191
		**p-value**	0.393	0.165	0.635	0.156	0.111	0.566	0.143	0.867	0.467	0.69	0.461	0.615	0.213	0.148	0.25
**Cerebellum**	**Lobule I-IV**	**rho**	0.066	-0.1	-0.001	-0.094	-0.179	-0.08	-0.134	-0.012	-0.112	-0.11	0.014	0.068	-0.19	-0.176	0.223
		**p-value**	0.691	0.546	0.996	0.568	0.276	0.627	0.415	0.941	0.498	0.504	0.933	0.685	0.252	0.29	0.179
	**Lobule V**	**rho**	0.116	0.051	0.029	0.133	-0.098	-0.036	-0.132	-0.104	-0.122	-0.089	0.165	0.082	-0.204	-0.31	0.287
		**p-value**	0.481	0.758	0.859	0.42	0.551	0.825	0.422	0.527	0.458	0.588	0.323	0.624	0.22	0.059	0.08
	**Lobule VI**	**rho**	0.132	0.087	0.222	0.216	-0.042	0.08	-0.089	-0.084	0.055	-0.09	0.186	-0.062	-0.202	**-0.422**	0.242
		**p-value**	0.423	0.598	0.174	0.186	0.798	0.629	0.591	0.61	0.738	0.585	0.263	0.711	0.225	**0.008**	0.144
	**Lobule VIIa-CrusI**	**rho**	0.053	-0.001	0.22	-0.031	-0.071	0.087	0.107	-0.203	-0.027	-0.059	0.258	0.005	0.041	-0.174	0.233
		**p-value**	0.749	0.994	0.178	0.853	0.666	0.596	0.517	0.215	0.87	0.719	0.118	0.975	0.808	0.297	0.16
	**Lobule VIIa-CrusII**	**rho**	-0.052	0.027	0.115	-0.049	0.205	-0.125	0.061	0.141	-0.149	0.039	-0.053	0.112	0.009	-0.208	-0.031
		**p-value**	0.755	0.87	0.485	0.766	0.21	0.449	0.714	0.391	0.366	0.816	0.751	0.502	0.957	0.21	0.854
	**Lobule VIIb**	**rho**	-0.017	0.013	0.021	0.062	0.249	-0.15	-0.034	0.028	-0.126	-0.061	0.02	0.257	0.056	-0.106	0.092
		**p-value**	0.919	0.936	0.899	0.706	0.126	0.361	0.838	0.868	0.446	0.713	0.906	0.119	0.74	0.526	0.583
	**Lobule VIIIa**	**rho**	-0.108	-0.114	0.009	-0.105	0.182	-0.129	-0.118	-0.121	-0.239	-0.233	-0.079	0.22	0.127	-0.111	0.104
		**p-value**	0.512	0.488	0.959	0.523	0.267	0.434	0.475	0.463	0.143	0.153	0.636	0.184	0.447	0.508	0.533
		**rho**	-0.134	-0.211	-0.152	-0.255	0.068	-0.211	-0.125	-0.206	-0.181	-0.174	-0.073	0.167	-0.038	-0.043	-0.003

	**Lobule VIIIb**	**p-value**	0.415	0.196	0.356	0.118	0.679	0.197	0.447	0.207	0.271	0.289	0.665	0.318	0.822	0.798	0.986
	**Lobule IX**	**rho**	0.024	-0.07	0.02	-0.019	0.097	-0.14	-0.206	-0.166	-0.021	-0.247	0.111	0.034	-0.135	-0.178	0.253
		**p-value**	0.885	0.674	0.905	0.911	0.557	0.397	0.208	0.313	0.9	0.129	0.507	0.842	0.42	0.285	0.125
	**Lobule X**	**rho**	0.132	0.227	0.216	0.133	0.237	0.031	0.24	-0.155	0.017	0.046	-0.173	-0.218	-0.226	**-0.446**	-0.264
		**p-value**	0.425	0.164	0.187	0.419	0.147	0.849	0.142	0.345	0.917	0.78	0.3	0.188	0.173	**0.005**	0.109
	**Dentate nuclei**	**rho**	0.144	0.264	0.134	0.063	-0.163	0.114	0.164	-0.037	0.07	0.113	0.151	-0.004	-0.059	-0.41	0.085
		**p-value**	0.382	0.104	0.417	0.702	0.322	0.491	0.32	0.824	0.672	0.494	0.366	0.98	0.723	0.011	0.613
	**Interposed nuclei**	**rho**	0.242	0.154	0.169	-0.059	-0.364	0.076	0.122	0.158	-0.039	0.159	-0.021	-0.11	-0.249	-0.2	0.051
		**p-value**	0.138	0.349	0.304	0.72	0.023	0.644	0.458	0.338	0.814	0.334	0.903	0.512	0.132	0.227	0.759
	**Fastigial nuclei**	**rho**	0.073	-0.062	0.256	0.011	-0.283	0.189	0.213	-0.079	-0.016	0.106	-0.098	-0.025	-0.139	-0.117	-0.145
		**p-value**	0.659	0.706	0.116	0.947	0.081	0.25	0.194	0.634	0.923	0.521	0.557	0.883	0.405	0.486	0.384
	**Whole cerebellum**	**rho**	0.032	-0.019	0.137	-0.033	0.083	-0.128	-0.063	-0.083	-0.151	-0.155	0.116	0.149	-0.068	-0.307	0.208
		**p-value**	0.849	0.909	0.404	0.844	0.614	0.436	0.702	0.616	0.358	0.346	0.487	0.373	0.687	0.061	0.211
	**ALS (n=52)**		**Memory**	**Everyday skills**	**Self-care skills**	**Mood changes**	**Odd beliefs**	**Abnormal behaviours**	**Eating habits**	**Sleep**	**Stereotypic and motor behaviours**	**Reduced motivation**	**Attention**	**Memory**	**Fluency**	**Language**	**Visuospatial**
**Thalamus**	**AV**	**rho**	0.234	0.002	0.003	0.063	0.238	0.072	0.253	0.148	0.189	0.072	0.098	0.212	-0.118	0.232	-0.119
		**p-value**	0.095	0.988	0.984	0.655	0.089	0.613	0.073	0.306	0.184	0.613	0.576	0.223	0.501	0.181	0.496
	**LD**	**rho**	-0.15	-0.16	-0.093	-0.22	-0.117	-0.092	-0.282	0.046	-0.264	-0.339	-0.086	0.237	0.097	0.34	-0.331
		**p-value**	0.288	0.266	0.518	0.117	0.41	0.515	0.045	0.749	0.062	0.014	0.623	0.171	0.579	0.045	0.052
	**LP**	**rho**	-0.029	0.063	0.093	-0.114	0.098	-0.121	-0.105	0.149	-0.101	-0.247	0.014	-0.029	0.025	0.294	-0.273
		**p-value**	0.84	0.664	0.516	0.421	0.49	0.392	0.462	0.302	0.482	0.078	0.937	0.868	0.889	0.087	0.113
	**VA**	**rho**	0.163	0.052	0.031	0.079	0.117	0.144	0.125	-0.155	0.105	0.173	0.152	-0.141	-0.076	-0.206	0.032
		**p-value**	0.247	0.721	0.831	0.58	0.41	0.307	0.38	0.283	0.464	0.22	0.384	0.42	0.666	0.236	0.855
	**VLa**	**rho**	0.178	0.116	0.084	0.106	0.098	0.141	0.238	-0.068	0.1	**0.362**	0.108	-0.282	-0.095	-0.318	0.256
		**p-value**	0.207	0.421	0.56	0.453	0.49	0.32	0.092	0.641	0.484	**0.008**	0.535	0.101	0.588	0.063	0.138
	**VLp**	**rho**	0.248	0.181	0.186	0.145	0.061	0.205	0.251	0.013	0.142	**0.404**	0.165	-0.33	-0.041	-0.187	0.351
		**p-value**	0.076	0.209	0.192	0.304	0.669	0.144	0.076	0.926	0.32	**0.003**	0.344	0.053	0.817	0.283	0.038
	**VPL**	**rho**	0.209	0.138	0.179	0.089	0.005	0.135	0.145	0.033	0.176	0.305	0.304	-0.124	-0.099	-0.201	0.334

		**p-value**	0.138	0.34	0.209	0.53	0.974	0.339	0.31	0.818	0.216	0.028	0.076	0.478	0.571	0.246	0.05
	**VM**	**rho**	0.169	0.083	0.132	0.061	-0.005	0.046	0.019	-0.048	0.118	0.202	0.33	-0.032	-0.09	-0.105	0.268
		**p-value**	0.23	0.567	0.354	0.67	0.974	0.748	0.893	0.741	0.408	0.151	0.053	0.857	0.607	0.549	0.12
	**Intralaminar**	**rho**	0.315	0.092	0.257	0.142	0.098	0.151	0.006	0.053	0.109	0.108	0.387	-0.023	-0.125	0.053	0.054
		**p-value**	0.023	0.525	0.068	0.316	0.49	0.284	0.967	0.716	0.448	0.447	0.022	0.894	0.473	0.761	0.758
	**Midline**	**rho**	0.263	0.065	0.22	0.075	0.135	0.111	-0.032	0.019	-0.01	-0.027	0.201	-0.049	-0.09	0.018	-0.138
		**p-value**	0.06	0.652	0.121	0.599	0.339	0.434	0.822	0.895	0.944	0.85	0.247	0.78	0.606	0.919	0.429
	**MD**	**rho**	-0.049	0.046	0.035	-0.118	0.07	-0.085	-0.111	0.07	-0.092	-0.096	-0.014	-0.118	-0.088	0.095	-0.297
		**p-value**	0.729	0.752	0.807	0.403	0.622	0.548	0.439	0.629	0.52	0.499	0.937	0.501	0.613	0.586	0.083
	**LGN**	**rho**	-0.035	-0.093	0.036	-0.187	-0.229	-0.219	-0.182	-0.109	-0.16	-0.296	0.414	0.23	0.002	0.154	0.158
		**p-value**	0.804	0.519	0.803	0.185	0.103	0.118	0.201	0.452	0.263	0.033	0.013	0.185	0.99	0.378	0.363
	**MGN**	**rho**	0.174	0.108	0.12	0.008	-0.07	-0.052	0.122	0.019	0.009	0.094	0.393	-0.005	-0.416	-0.052	0.241
		**p-value**	0.217	0.455	0.403	0.954	0.622	0.714	0.392	0.897	0.948	0.507	0.02	0.976	0.013	0.767	0.164
	**Pulvinar**	**rho**	-0.001	0.031	0.173	-0.079	0.023	-0.059	-0.101	0.075	-0.009	-0.163	0.196	0.287	0.07	0.122	0.125
		**p-value**	0.997	0.828	0.224	0.578	0.87	0.677	0.479	0.604	0.951	0.249	0.26	0.095	0.688	0.484	0.475
	**Whole thalamus**	**rho**	0.18	0.131	0.209	0.037	0.051	0.061	0.05	0.056	0.11	0.119	0.341	0.022	-0.087	0.024	0.14
		**p-value**	0.202	0.365	0.141	0.794	0.718	0.666	0.726	0.697	0.442	0.403	0.045	0.898	0.619	0.89	0.423
**Cerebellum**	**Lobule I-IV**	**rho**	0.213	0.35	**0.365**	0.166	-0.005	0.253	**0.299**	0.252	0.29	**0.394**	-0.278	-0.268	0.031	0.039	0.104
		**p-value**	0.129	0.013	**0.009**	0.24	0.974	0.071	**0.033**	0.077	0.039	**0.004**	0.106	0.119	0.861	0.824	0.552
	**Lobule V**	**rho**	0.274	0.312	0.263	0.246	0.135	0.072	**0.397**	0.261	0.327	0.346	-0.004	-0.043	-0.261	0.195	-0.032
		**p-value**	0.05	0.027	0.062	0.079	0.339	0.614	**0.004**	0.067	0.019	0.012	0.984	0.808	0.13	0.262	0.856
	**Lobule VI**	**rho**	0.216	**0.416**	0.348	0.231	0.079	0.128	0.093	0.297	0.128	0.338	-0.13	-0.029	0.155	0.226	-0.01
		**p-value**	0.124	**0.003**	0.012	0.1	0.576	0.367	0.518	0.036	0.372	0.014	0.457	0.871	0.373	0.192	0.952
	**Lobule VIIa-CrusI**	**rho**	0.168	0.144	0.006	0.131	-0.005	-0.013	0.139	0.056	0.044	0.264	0.164	-0.028	-0.224	0.129	0.115
		**p-value**	0.234	0.319	0.966	0.353	0.974	0.925	0.33	0.702	0.761	0.059	0.348	0.873	0.196	0.462	0.512
	**Lobule VIIa-CrusII**	**rho**	0.106	0.149	0.102	0.084	0.191	0.135	0.164	0.01	0.256	0.336	0.036	-0.091	-0.32	0.004	0.193
		**p-value**	0.455	0.301	0.476	0.552	0.174	0.339	0.251	0.946	0.07	0.015	0.835	0.603	0.061	0.982	0.266
	**Lobule VIIb**	**rho**	0.1	0.31	0.278	0.235	0.098	0.165	0.159	0.215	0.344	0.309	-0.193	-0.088	-0.12	0.085	0.135

		**p-value**	0.482	0.028	0.048	0.093	0.49	0.243	0.264	0.133	0.013	0.026	0.266	0.615	0.491	0.629	0.44
	**Lobule VIIIa**	**rho**	0.102	0.141	0.16	0.099	0.033	0.099	0.075	0.229	0.359	0.098	-0.222	0.012	-0.053	0.337	-0.05
		**p-value**	0.471	0.33	0.262	0.483	0.818	0.485	0.602	0.11	0.01	0.488	0.199	0.944	0.763	0.047	0.775
	**Lobule VIIIb**	**rho**	-0.1	-0.076	-0.003	-0.15	-0.042	-0.127	-0.116	-0.027	0.076	-0.216	-0.2	0.213	-0.042	0.351	-0.089
		**p-value**	0.479	0.598	0.986	0.289	0.768	0.368	0.416	0.855	0.597	0.124	0.248	0.22	0.81	0.039	0.61
	**Lobule IX**	**rho**	0.058	0.163	0.077	0.023	0.014	-0.066	0.147	0.071	0.166	0.023	-0.136	-0.028	-0.265	0.326	0.149
		**p-value**	0.685	0.258	0.593	0.869	0.922	0.642	0.302	0.624	0.245	0.871	0.436	0.875	0.124	0.056	0.392
	**Lobule X**	**rho**	-0.016	0.023	0.024	0.009	0.173	-0.068	0.131	0.121	0.249	0.095	-0.162	0.026	-0.081	0.176	-0.217
		**p-value**	0.913	0.874	0.865	0.949	0.221	0.633	0.358	0.401	0.078	0.502	0.352	0.883	0.643	0.312	0.21
	**Dentate nuclei**	**rho**	0.283	**0.373**	0.323	0.327	0.154	0.212	0.118	0.129	0.295	0.237	0.055	0.098	-0.168	0.209	0.087
		**p-value**	0.042	**0.008**	0.021	0.018	0.276	0.132	0.409	0.371	0.036	0.091	0.755	0.575	0.333	0.229	0.617
	**Interposed nuclei**	**rho**	0.177	0.066	0.032	0.04	-0.079	0.186	0.058	0.053	0.181	0.199	0.164	-0.004	-0.023	-0.005	-0.142
		**p-value**	0.209	0.648	0.822	0.78	0.576	0.186	0.685	0.717	0.203	0.158	0.347	0.981	0.894	0.977	0.415
	**Fastigial nuclei**	**rho**	0.031	-0.083	-0.161	-0.031	0.089	0.224	0.229	0.02	0.238	0.274	-0.1	-0.273	-0.283	-0.327	-0.14
		**p-value**	0.828	0.568	0.259	0.829	0.532	0.11	0.106	0.891	0.092	0.05	0.569	0.113	0.1	0.055	0.422
	**Whole cerebellum**	**rho**	0.199	0.297	0.194	0.219	0.145	0.126	0.278	0.244	0.341	**0.367**	-0.171	-0.011	-0.159	0.216	0.016
		**p-value**	0.158	0.036	0.171	0.118	0.306	0.375	0.049	0.088	0.014	**0.008**	0.326	0.951	0.361	0.212	0.928
	**bvFTD sporadic (n=41)**		**Memory**	**Everyday skills**	**Self-care skills**	**Mood changes**	**Odd beliefs**	**Abnormal behaviours**	**Eating habits**	**Sleep**	**Stereotypic and motor behaviours**	**Reduced motivation**	**Attention**	**Memory**	**Fluency**	**Language**	**Visuospatial**
**Thalamus**	**AV**	**rho**	0.039	-0.261	**-0.429**	0.155	-0.033	-0.046	-0.311	-0.262	-0.089	-0.306	-0.04	0.079	0.377	0.334	0.226
		**p-value**	0.807	0.099	**0.005**	0.332	0.837	0.775	0.048	0.098	0.578	0.052	0.805	0.623	0.015	0.033	0.155
	**LD**	**rho**	-0.001	-0.219	-0.339	0.132	0.119	-0.026	-0.091	-0.335	-0.023	-0.233	-0.089	-0.085	0.189	0.131	0.129
		**p-value**	0.996	0.169	0.03	0.411	0.458	0.873	0.573	0.032	0.888	0.143	0.582	0.595	0.237	0.413	0.42
	**LP**	**rho**	-0.148	-0.289	-0.221	0.068	-0.215	-0.13	-0.375	-0.336	-0.209	-0.284	0.048	0.058	0.319	0.285	-0.003
		**p-value**	0.355	0.067	0.164	0.672	0.177	0.417	0.016	0.032	0.19	0.072	0.768	0.718	0.042	0.071	0.984
	**VA**	**rho**	-0.03	-0.337	-0.293	0.139	0.016	-0.111	-0.319	-0.203	-0.117	-0.22	0.08	0.18	0.348	0.368	0.099
		**p-value**	0.85	0.031	0.063	0.385	0.922	0.488	0.042	0.204	0.468	0.167	0.619	0.259	0.026	0.018	0.536
	**VLa**	**rho**	-0.255	**-0.505**	-0.35	-0.137	-0.123	-0.286	**-0.48**	**-0.482**	-0.241	**-0.457**	0.169	0.035	0.375	0.277	0.107

		**p-value**	0.108	**0.001**	0.025	0.393	0.442	0.07	**0.001**	**0.001**	0.128	**0.003**	0.29	0.828	0.016	0.079	0.506
	**VLp**	**rho**	-0.276	**-0.453**	-0.321	-0.163	-0.075	-0.232	**-0.43**	**-0.409**	-0.254	**-0.403**	0.143	-0.045	0.304	0.268	0.118
		**p-value**	0.081	**0.003**	0.041	0.309	0.639	0.145	**0.005**	**0.008**	0.11	**0.009**	0.374	0.779	0.053	0.09	0.463
	**VPL**	**rho**	-0.113	-0.242	-0.178	-0.158	-0.135	-0.236	-0.309	-0.373	-0.113	-0.38	0.041	-0.15	-0.046	0.191	-0.064
		**p-value**	0.482	0.128	0.266	0.325	0.4	0.138	0.05	0.016	0.481	0.014	0.797	0.348	0.774	0.231	0.693
	**VM**	**rho**	-0.18	-0.161	-0.011	-0.113	-0.185	-0.183	-0.258	-0.282	-0.087	-0.25	0.057	-0.124	-0.064	0.152	-0.059
		**p-value**	0.259	0.313	0.946	0.481	0.248	0.251	0.104	0.074	0.588	0.115	0.723	0.438	0.692	0.344	0.713
	**Intralaminar**	**rho**	-0.169	-0.261	-0.191	0.038	-0.203	-0.035	-0.188	-0.233	-0.189	-0.202	0.104	-0.017	0.251	0.348	0.235
		**p-value**	0.291	0.099	0.232	0.813	0.204	0.828	0.24	0.142	0.236	0.205	0.518	0.918	0.114	0.026	0.139
	**Midline**	**rho**	-0.203	-0.24	-0.37	0.072	-0.053	-0.051	-0.222	-0.282	-0.176	-0.249	0.087	0.066	**0.42**	**0.451**	0.196
		**p-value**	0.202	0.131	0.017	0.656	0.743	0.753	0.163	0.074	0.271	0.116	0.588	0.681	**0.006**	**0.003**	0.219
	**MD**	**rho**	-0.206	-0.34	**-0.408**	0.035	-0.078	-0.036	-0.312	-0.327	-0.067	**-0.481**	0.14	0.231	**0.458**	0.358	0.071
		**p-value**	0.196	0.029	**0.008**	0.83	0.626	0.824	0.047	0.037	0.678	**0.001**	0.381	0.147	**0.003**	0.021	0.658
	**LGN**	**rho**	-0.209	0.035	0.162	0.135	-0.047	-0.045	0.138	0.151	0.008	0.083	0.13	**0.449**	-0.234	-0.14	-0.257
		**p-value**	0.189	0.828	0.311	0.4	0.768	0.778	0.391	0.346	0.959	0.607	0.418	**0.003**	0.142	0.383	0.104
	**MGN**	**rho**	-0.057	-0.312	-0.181	-0.109	0.112	-0.208	-0.035	-0.264	0.016	-0.279	0.043	-0.01	-0.061	0.306	0.023
		**p-value**	0.723	0.047	0.258	0.498	0.487	0.192	0.826	0.096	0.923	0.077	0.788	0.952	0.707	0.052	0.887
	**Pulvinar**	**rho**	-0.009	-0.218	-0.003	0.218	0.085	-0.193	-0.021	0.012	-0.028	-0.023	0.029	0.027	-0.16	-0.008	-0.026
		**p-value**	0.957	0.17	0.983	0.171	0.599	0.226	0.897	0.939	0.862	0.888	0.858	0.866	0.317	0.96	0.872
	**Whole thalamus**	**rho**	-0.238	**-0.49**	**-0.399**	0.021	-0.064	-0.216	-0.365	**-0.4**	-0.173	**-0.423**	0.113	0.117	0.311	0.34	0.067
		**p-value**	0.135	**0.001**	**0.01**	0.896	0.69	0.175	0.019	**0.009**	0.278	**0.006**	0.48	0.465	0.048	0.029	0.676
**Cerebellum**	**Lobule I-IV**	**rho**	-0.123	-0.088	-0.045	0.158	0.232	-0.161	-0.007	-0.263	0.022	0.173	-0.027	-0.244	-0.271	0.139	0.022
		**p-value**	0.444	0.582	0.782	0.322	0.145	0.314	0.965	0.096	0.891	0.278	0.867	0.123	0.087	0.387	0.891
	**Lobule V**	**rho**	0.114	-0.168	-0.373	0.059	0.037	-0.191	-0.225	-0.295	0.09	-0.217	-0.183	-0.162	-0.009	0.331	0.221
		**p-value**	0.479	0.293	0.016	0.714	0.818	0.233	0.157	0.061	0.575	0.173	0.252	0.311	0.958	0.034	0.165
	**Lobule VI**	**rho**	-0.125	0.004	-0.157	-0.023	-0.005	-0.162	-0.249	-0.126	0.167	-0.145	0.233	-0.082	0.333	0.209	0.219
		**p-value**	0.435	0.981	0.326	0.885	0.977	0.312	0.116	0.434	0.298	0.366	0.143	0.611	0.034	0.191	0.169
		**rho**	0.004	-0.211	-0.1	-0.169	-0.001	-0.356	-0.176	-0.22	-0.162	-0.296	-0.14	-0.272	0.035	0.368	-0.069

	**Lobule VIIa-CrusI**	**p-value**	0.98	0.185	0.533	0.291	0.995	0.022	0.271	0.166	0.312	0.061	0.384	0.085	0.829	0.018	0.667
	**Lobule VIIa-CrusII**	**rho**	-0.065	-0.235	-0.231	-0.19	-0.24	-0.279	-0.176	-0.254	0.004	-0.258	0.019	-0.328	0.026	0.051	0.135
		**p-value**	0.686	0.139	0.147	0.233	0.131	0.077	0.271	0.109	0.982	0.104	0.907	0.036	0.872	0.751	0.4
	**Lobule VIIb**	**rho**	-0.233	-0.12	-0.128	-0.102	-0.161	-0.273	-0.184	-0.364	-0.004	-0.059	0.049	-0.334	-0.072	0.15	0.081
		**p-value**	0.143	0.453	0.425	0.525	0.315	0.084	0.249	0.019	0.979	0.715	0.763	0.033	0.655	0.35	0.616
	**Lobule VIIIa**	**rho**	-0.201	-0.122	0.016	0.033	0.121	-0.167	-0.111	-0.326	0.015	0.051	0.183	-0.127	-0.162	0.032	0.075
		**p-value**	0.207	0.448	0.923	0.839	0.45	0.297	0.491	0.038	0.925	0.752	0.252	0.431	0.313	0.841	0.641
	**Lobule VIIIb**	**rho**	-0.042	-0.257	-0.176	0.12	0.384	0.086	-0.074	-0.19	0.06	0.128	-0.065	-0.07	-0.016	-0.09	-0.077
		**p-value**	0.794	0.105	0.271	0.456	0.013	0.595	0.647	0.233	0.708	0.425	0.686	0.662	0.922	0.575	0.633
	**Lobule IX**	**rho**	0.109	-0.153	-0.057	0.015	0.289	0.165	-0.042	0.04	0.141	0.25	0.091	-0.047	-0.129	-0.047	-0.039
		**p-value**	0.498	0.34	0.722	0.927	0.067	0.303	0.795	0.804	0.379	0.115	0.572	0.772	0.423	0.769	0.81
	**Lobule X**	**rho**	-0.134	-0.082	-0.083	0.001	0.095	-0.014	-0.074	-0.084	0.206	0.039	-0.053	-0.145	0.048	0.003	0.036
		**p-value**	0.405	0.609	0.605	0.997	0.555	0.932	0.644	0.601	0.197	0.807	0.742	0.366	0.767	0.986	0.825
	**Dentate nuclei**	**rho**	-0.125	-0.194	-0.238	0.021	0.217	-0.092	-0.202	-0.127	-0.059	-0.151	-0.109	-0.208	-0.147	-0.18	-0.259
		**p-value**	0.438	0.223	0.134	0.894	0.172	0.568	0.205	0.427	0.714	0.347	0.499	0.193	0.36	0.261	0.102
	**Interposed nuclei**	**rho**	-0.085	0.029	0.015	0.206	-0.091	-0.039	-0.008	0.051	-0.117	-0.045	0.062	-0.038	0.025	-0.076	-0.098
		**p-value**	0.596	0.855	0.924	0.196	0.573	0.808	0.961	0.751	0.466	0.78	0.701	0.814	0.878	0.636	0.541
	**Fastigial nuclei**	**rho**	-0.081	-0.004	0.069	0.266	-0.011	0.163	0.215	-0.01	0.094	0.076	0.025	0.273	0.04	-0.018	-0.131
		**p-value**	0.614	0.983	0.669	0.093	0.945	0.309	0.177	0.949	0.558	0.637	0.879	0.085	0.803	0.909	0.413
	**Whole cerebellum**	**rho**	-0.102	-0.21	-0.288	-0.066	0.071	-0.25	-0.248	-0.361	0.026	-0.177	-0.002	-0.349	0.058	0.236	0.044
		**p-value**	0.526	0.187	0.068	0.68	0.659	0.115	0.118	0.02	0.874	0.268	0.992	0.026	0.718	0.138	0.784
	**bvFTD *C9orf72* (n=12)**		**Memory**	**Everyday skills**	**Self-care skills**	**Mood changes**	**Odd beliefs**	**Abnormal behaviours**	**Eating habits**	**Sleep**	**Stereotypic and motor behaviours**	**Reduced motivation**	**Attention**	**Memory**	**Fluency**	**Language**	**Visuospatial**
**Thalamus**	**AV**	**rho**	-0.643	-0.682	-0.655	-0.077	-0.355	-0.309	-0.618	0.199	0.014	-0.172	0.201	0.541	0.638	0.499	0.388
		**p-value**	0.024	0.015	0.021	0.811	0.258	0.328	0.032	0.534	0.965	0.593	0.53	0.069	0.025	0.099	0.213
	**LD**	**rho**	-0.415	-0.343	-0.048	0.636	0.19	0.25	-0.201	-0.263	0.127	-0.133	0.329	0.228	0.418	0.197	0.306
		**p-value**	0.18	0.275	0.883	0.026	0.554	0.434	0.53	0.408	0.694	0.68	0.297	0.475	0.176	0.54	0.333
	**LP**	**rho**	-0.671	-0.449	-0.662	-0.341	-0.419	-0.475	-0.583	0.256	-0.134	-0.407	0.413	0.295	0.511	0.278	0.438

		**p-value**	0.017	0.143	0.019	0.278	0.175	0.119	0.047	0.422	0.677	0.189	0.182	0.351	0.09	0.382	0.155
	**VA**	**rho**	-0.583	**-0.763**	-0.662	-0.531	**-0.71**	**-0.717**	-0.636	0.056	-0.378	-0.425	0.233	0.707	0.631	**0.721**	0.37
		**p-value**	0.046	**0.004**	0.019	0.076	**0.01**	**0.009**	0.026	0.862	0.226	0.169	0.466	0.01	0.028	**0.008**	0.236
	**VLa**	**rho**	-0.366	-0.555	-0.575	**-0.735**	**-0.835**	**-0.815**	-0.562	-0.049	-0.601	-0.33	0.145	0.531	0.454	0.583	0.221
		**p-value**	0.243	0.061	0.051	**0.007**	**0.001**	**0.001**	0.057	0.88	0.039	0.295	0.653	0.076	0.138	0.046	0.491
	**VLp**	**rho**	-0.334	-0.555	-0.527	-0.699	**-0.85**	**-0.801**	-0.569	-0.064	-0.64	-0.404	0.177	0.569	0.454	0.622	0.206
		**p-value**	0.289	0.061	0.078	0.011	**0**	**0.002**	0.054	0.843	0.025	0.193	0.583	0.053	0.138	0.031	0.52
	**VPL**	**rho**	-0.084	-0.184	-0.242	-0.32	-0.566	-0.471	-0.424	-0.12	-0.587	-0.27	0.014	0.359	0.241	0.306	0.149
		**p-value**	0.794	0.568	0.449	0.311	0.055	0.122	0.169	0.709	0.045	0.396	0.965	0.252	0.45	0.334	0.643
	**VM**	**rho**	-0.485	-0.477	-0.615	-0.411	-0.706	**-0.738**	-0.693	-0.15	-0.636	-0.225	0.12	0.475	0.56	0.436	0.42
		**p-value**	0.11	0.117	0.033	0.184	0.01	**0.006**	0.013	0.641	0.026	0.483	0.71	0.119	0.058	0.157	0.174
	**Intralaminar**	**rho**	**-0.735**	-0.375	**-0.747**	-0.443	-0.391	-0.573	-0.505	0.162	-0.368	-0.295	0.428	0.278	0.546	0.271	0.655
		**p-value**	**0.007**	0.23	**0.005**	0.149	0.209	0.052	0.094	0.615	0.24	0.352	0.166	0.382	0.066	0.395	0.021
	**Midline**	**rho**	**-0.826**	**-0.792**	-0.604	0.109	-0.341	-0.429	-0.686	-0.049	-0.265	-0.309	0.481	**0.735**	**0.915**	**0.714**	0.705
		**p-value**	**0.001**	**0.002**	0.038	0.736	0.279	0.164	0.014	0.88	0.405	0.329	0.114	**0.007**	**0**	**0.009**	0.01
	**MD**	**rho**	-0.696	**-0.838**	**-0.802**	-0.436	**-0.774**	**-0.773**	**-0.77**	-0.237	-0.65	-0.326	0.18	0.527	**0.723**	0.671	0.292
		**p-value**	0.012	**0.001**	**0.002**	0.157	**0.003**	**0.003**	**0.003**	0.458	0.022	0.301	0.575	0.078	**0.008**	0.017	0.357
	**LGN**	**rho**	0.32	0.18	0.081	-0.183	-0.208	0.067	0.035	-0.015	-0.304	-0.319	-0.039	-0.19	-0.333	-0.081	-0.424
		**p-value**	0.311	0.575	0.804	0.57	0.517	0.837	0.913	0.963	0.337	0.312	0.905	0.555	0.29	0.803	0.17
	**MGN**	**rho**	0.657	0.449	0.384	0.081	0.079	0.257	0.311	-0.226	-0.198	0.175	-0.417	-0.471	-0.596	-0.355	-0.641
		**p-value**	0.02	0.143	0.217	0.803	0.807	0.421	0.325	0.481	0.538	0.585	0.177	0.122	0.041	0.257	0.025
	**Pulvinar**	**rho**	-0.169	-0.417	-0.073	0.33	-0.366	-0.042	-0.488	-0.331	-0.258	-0.467	0.346	0.253	0.298	0.334	-0.121
		**p-value**	0.6	0.177	0.821	0.294	0.242	0.896	0.108	0.293	0.418	0.126	0.27	0.427	0.347	0.289	0.708
	**Whole thalamus**	**rho**	-0.615	**-0.806**	-0.692	-0.411	**-0.882**	**-0.728**	**-0.809**	-0.222	-0.64	-0.628	0.396	0.654	0.653	**0.714**	0.306
		**p-value**	0.033	**0.002**	0.013	0.184	**0**	**0.007**	**0.001**	0.488	0.025	0.029	0.203	0.021	0.021	**0.009**	0.333
**Cerebellum**	**Lobule I-IV**	**rho**	0.232	0.113	0.238	-0.306	-0.093	0.004	0.18	0.214	-0.102	0.088	0.138	0.169	0.071	0.225	-0.043
		**p-value**	0.468	0.726	0.457	0.334	0.773	0.991	0.575	0.503	0.751	0.786	0.669	0.6	0.827	0.482	0.895
	**Lobule V**	**rho**	0.387	0.205	0.399	-0.183	-0.262	0.13	0.113	-0.026	-0.177	-0.284	0.339	0.091	-0.142	0.098	-0.231

		**p-value**	0.214	0.523	0.199	0.57	0.411	0.687	0.726	0.935	0.583	0.371	0.281	0.778	0.66	0.761	0.469
	**Lobule VI**	**rho**	-0.112	-0.092	0.062	-0.548	-0.441	-0.383	-0.049	0.053	-0.219	**-0.751**	0.664	0.422	0.106	0.33	0.317
		**p-value**	0.728	0.776	0.848	0.065	0.151	0.219	0.879	0.871	0.494	**0.005**	0.018	0.172	0.742	0.294	0.316
	**Lobule VIIa-CrusI**	**rho**	0.053	0.254	0.059	-0.464	-0.355	-0.243	0.014	-0.211	-0.322	-0.382	0.329	-0.144	-0.192	-0.207	0.061
		**p-value**	0.871	0.425	0.857	0.129	0.258	0.448	0.965	0.511	0.308	0.22	0.297	0.655	0.551	0.518	0.852
	**Lobule VIIa-CrusII**	**rho**	0.011	-0.11	0.432	0.552	0.258	0.218	0.194	-0.421	0.148	-0.316	0.247	0.327	0.021	0.186	0.231
		**p-value**	0.974	0.735	0.161	0.063	0.418	0.496	0.545	0.172	0.645	0.317	0.438	0.3	0.948	0.562	0.469
	**Lobule VIIb**	**rho**	-0.105	-0.163	0.388	0.51	0.201	0.295	0.11	-0.226	0.201	-0.572	0.544	0.394	0.135	0.271	0.267
		**p-value**	0.744	0.614	0.213	0.091	0.532	0.351	0.735	0.481	0.53	0.052	0.067	0.205	0.676	0.395	0.402
	**Lobule VIIIa**	**rho**	-0.369	-0.138	0.062	0.236	0.061	0.077	-0.039	-0.233	0.113	-0.579	0.576	0.211	0.17	0.06	0.431
		**p-value**	0.238	0.669	0.848	0.461	0.851	0.811	0.905	0.466	0.726	0.049	0.05	0.511	0.597	0.854	0.162
	**Lobule VIIIb**	**rho**	-0.643	-0.219	-0.395	-0.591	-0.176	-0.612	0.021	-0.214	-0.307	-0.446	0.509	0.162	0.248	0.144	**0.733**
		**p-value**	0.024	0.494	0.203	0.043	0.585	0.035	0.948	0.503	0.331	0.147	0.091	0.616	0.437	0.655	**0.007**
	**Lobule IX**	**rho**	-0.218	-0.032	-0.128	-0.362	-0.165	-0.517	-0.018	-0.079	-0.053	-0.305	0.124	0.281	-0.014	0.028	0.552
		**p-value**	0.496	0.922	0.692	0.247	0.608	0.085	0.957	0.807	0.87	0.335	0.702	0.376	0.965	0.931	0.063
	**Lobule X**	**rho**	-0.123	0.201	0	-0.109	0.082	-0.274	0.152	-0.354	-0.23	-0.147	-0.028	-0.148	-0.163	-0.264	0.349
		**p-value**	0.703	0.53	1	0.736	0.799	0.388	0.637	0.259	0.473	0.648	0.931	0.647	0.612	0.408	0.266
	**Dentate nuclei**	**rho**	-0.341	-0.59	-0.285	0.25	-0.534	-0.204	**-0.71**	-0.23	-0.382	-0.365	0.368	0.626	0.617	0.598	0.214
		**p-value**	0.278	0.043	0.368	0.434	0.074	0.525	**0.01**	0.473	0.221	0.243	0.24	0.03	0.033	0.04	0.505
	**Interposed nuclei**	**rho**	0.162	-0.078	-0.19	-0.218	-0.62	-0.211	-0.512	0.026	-0.484	-0.007	-0.127	0.165	0.156	0.179	-0.256
		**p-value**	0.616	0.81	0.554	0.496	0.031	0.511	0.089	0.935	0.111	0.983	0.694	0.608	0.628	0.577	0.421
	**Fastigial nuclei**	**rho**	0.52	0.452	0.34	-0.288	-0.072	0.112	0.3	-0.158	0.124	0.253	-0.428	-0.415	-0.553	-0.496	-0.491
		**p-value**	0.083	0.14	0.279	0.364	0.825	0.728	0.343	0.624	0.702	0.428	0.166	0.18	0.062	0.101	0.105
	**Whole cerebellum**	**rho**	-0.13	-0.032	0.044	-0.285	-0.405	-0.267	-0.131	-0.245	-0.247	-0.639	0.576	0.228	0.043	0.098	0.274
		**p-value**	0.687	0.922	0.892	0.37	0.191	0.401	0.685	0.444	0.438	0.025	0.05	0.475	0.896	0.761	0.389
	**ALS-FTD sporadic (n=29)**		**Memory**	**Everyday skills**	**Self-care skills**	**Mood changes**	**Odd beliefs**	**Abnormal behaviours**	**Eating habits**	**Sleep**	**Stereotypic and motor behaviours**	**Reduced motivation**	**Attention**	**Memory**	**Fluency**	**Language**	**Visuospatial**
**Thalamus**	**AV**	**rho**	0.063	-0.024	-0.181	0.107	-0.178	0.04	-0.127	0.001	0.022	-0.053	0.076	-0.004	0.163	-0.167	0.067

		**p-value**	0.756	0.904	0.365	0.595	0.376	0.844	0.529	0.996	0.912	0.791	0.7	0.986	0.408	0.396	0.736
	**LD**	**rho**	-0.055	0.129	0.149	-0.064	-0.1	0.184	0.085	0.036	-0.019	0.021	-0.394	-0.099	-0.383	-0.397	-0.384
		**p-value**	0.784	0.522	0.457	0.753	0.62	0.357	0.672	0.858	0.923	0.915	0.038	0.617	0.044	0.037	0.044
	**LP**	**rho**	0.46	0.343	0.204	0.164	-0.25	0.33	0.211	0.307	0.216	0.174	-0.097	-0.314	-0.263	-0.382	-0.054
		**p-value**	0.016	0.08	0.307	0.413	0.208	0.093	0.292	0.12	0.28	0.385	0.622	0.104	0.177	0.045	0.784
	**VA**	**rho**	-0.171	-0.097	0.097	-0.042	**-0.532**	-0.1	-0.294	-0.357	-0.275	-0.313	0.108	0.007	0.159	-0.149	0.114
		**p-value**	0.394	0.63	0.632	0.834	**0.004**	0.62	0.137	0.068	0.165	0.111	0.585	0.971	0.418	0.449	0.564
	**VLa**	**rho**	0.287	0.251	0.283	0.266	-0.34	0.106	0.068	-0.102	-0.031	0.052	-0.056	-0.215	-0.159	-0.383	0.095
		**p-value**	0.146	0.206	0.153	0.18	0.083	0.599	0.736	0.614	0.878	0.795	0.775	0.272	0.419	0.044	0.632
	**VLp**	**rho**	0.446	0.359	0.307	0.389	-0.072	0.089	0.178	0.051	0.059	0.186	-0.002	-0.203	-0.259	-0.477	0.05
		**p-value**	0.02	0.066	0.119	0.045	0.72	0.658	0.376	0.801	0.77	0.354	0.99	0.3	0.184	0.01	0.801
	**VPL**	**rho**	0.431	0.125	0.158	0.188	0.131	-0.055	0.113	0.093	-0.18	0.361	0.049	-0.154	-0.136	-0.159	0.064
		**p-value**	0.025	0.536	0.432	0.347	0.515	0.787	0.575	0.645	0.368	0.065	0.806	0.433	0.49	0.42	0.748
	**VM**	**rho**	0.418	0.083	0.189	0.228	0.056	0.05	0.102	0.157	-0.152	0.297	0.024	-0.116	-0.091	-0.166	0.161
		**p-value**	0.03	0.681	0.345	0.253	0.78	0.806	0.611	0.434	0.449	0.132	0.902	0.555	0.646	0.399	0.414
	**Intralaminar**	**rho**	0.169	0.219	0.128	0.116	-0.1	0.113	-0.039	-0.069	-0.218	0.054	-0.151	-0.068	0.067	-0.403	0.008
		**p-value**	0.401	0.272	0.523	0.566	0.619	0.574	0.848	0.731	0.276	0.79	0.445	0.731	0.736	0.034	0.968
	**Midline**	**rho**	0.014	-0.147	0.039	-0.023	-0.175	0.012	-0.36	-0.207	-0.193	-0.122	0.009	0	0.313	-0.258	0.184
		**p-value**	0.946	0.463	0.845	0.911	0.382	0.953	0.065	0.299	0.334	0.546	0.964	1	0.105	0.185	0.349
	**MD**	**rho**	0.046	-0.102	-0.062	0.058	-0.241	-0.059	-0.394	-0.218	-0.098	-0.182	0.186	0.12	0.267	-0.146	0.322
		**p-value**	0.821	0.611	0.758	0.775	0.225	0.771	0.042	0.275	0.627	0.363	0.343	0.542	0.169	0.457	0.094
	**LGN**	**rho**	0.104	-0.102	-0.092	0.232	-0.181	-0.173	-0.164	-0.016	0.001	-0.197	0.258	-0.02	0.111	0.148	0.051
		**p-value**	0.606	0.612	0.647	0.244	0.366	0.387	0.414	0.937	0.996	0.324	0.185	0.919	0.574	0.452	0.795
	**MGN**	**rho**	0.288	0.026	-0.007	-0.087	-0.099	-0.218	-0.318	0.012	-0.347	0.046	0.313	0.049	0.125	-0.068	0.203
		**p-value**	0.145	0.897	0.973	0.666	0.622	0.274	0.106	0.954	0.076	0.82	0.105	0.805	0.525	0.729	0.301
	**Pulvinar**	**rho**	0.208	0.292	0.173	0.255	-0.3	0.172	-0.01	-0.034	0.109	0.03	0.002	-0.052	-0.259	-0.313	0.092
		**p-value**	0.299	0.139	0.388	0.199	0.129	0.39	0.961	0.866	0.588	0.88	0.99	0.794	0.183	0.105	0.643
		**rho**	0.318	0.217	0.162	0.269	-0.321	0.129	-0.097	-0.074	-0.009	0.032	0.065	-0.087	-0.102	-0.321	0.156

	**Whole thalamus**	**p-value**	0.106	0.277	0.419	0.175	0.103	0.52	0.63	0.715	0.963	0.876	0.743	0.661	0.605	0.095	0.427
**Cerebellum**	**Lobule I-IV**	**rho**	0.159	0.085	0.185	0.049	-0.089	0.026	-0.123	0.099	-0.107	-0.029	0.173	0.026	-0.143	-0.229	0.416
		**p-value**	0.428	0.675	0.356	0.808	0.66	0.899	0.542	0.623	0.596	0.886	0.38	0.896	0.468	0.242	0.028
	**Lobule V**	**rho**	0.25	0.043	0.138	0.204	-0.094	-0.018	-0.099	0.012	-0.12	0.032	0.316	0.055	-0.129	-0.335	0.427
		**p-value**	0.209	0.829	0.493	0.307	0.643	0.929	0.625	0.953	0.549	0.874	0.102	0.783	0.512	0.082	0.023
	**Lobule VI**	**rho**	0.069	-0.006	0.286	0.151	-0.258	-0.124	-0.127	0.005	0.036	-0.056	0.175	-0.107	-0.272	-0.394	0.263
		**p-value**	0.731	0.976	0.148	0.454	0.194	0.539	0.528	0.98	0.858	0.78	0.374	0.586	0.161	0.038	0.176
	**Lobule VIIa-CrusI**	**rho**	0.052	-0.012	0.144	-0.104	-0.134	-0.102	-0.018	-0.122	-0.082	0.028	0.146	0.012	-0.223	-0.226	0.226
		**p-value**	0.798	0.954	0.472	0.606	0.506	0.611	0.928	0.544	0.686	0.891	0.458	0.952	0.255	0.248	0.247
	**Lobule VIIa-CrusII**	**rho**	0.013	0.119	0.139	0.056	0.41	-0.044	0.148	0.157	-0.088	0.144	-0.057	0.09	-0.01	-0.205	0.015
		**p-value**	0.95	0.554	0.491	0.781	0.034	0.828	0.461	0.434	0.661	0.475	0.772	0.649	0.959	0.295	0.938
	**Lobule VIIb**	**rho**	0.016	-0.026	-0.069	0.09	0.26	-0.213	-0.033	0.119	-0.157	0.095	0.115	0.277	0.154	-0.046	0.172
		**p-value**	0.937	0.897	0.733	0.656	0.191	0.285	0.869	0.553	0.435	0.639	0.56	0.153	0.435	0.817	0.381
	**Lobule VIIIa**	**rho**	-0.198	-0.172	-0.126	-0.138	0.051	-0.226	-0.203	-0.05	-0.354	-0.079	0.055	0.278	0.206	-0.017	0.24
		**p-value**	0.322	0.391	0.531	0.494	0.8	0.258	0.309	0.806	0.07	0.695	0.781	0.152	0.294	0.931	0.218
	**Lobule VIIIb**	**rho**	-0.21	-0.294	-0.196	-0.303	0.006	-0.311	-0.171	-0.255	-0.218	-0.115	0.03	0.185	-0.003	0.053	0.057
		**p-value**	0.294	0.137	0.328	0.125	0.975	0.114	0.394	0.199	0.274	0.568	0.881	0.346	0.989	0.79	0.774
	**Lobule IX**	**rho**	-0.018	-0.222	-0.075	-0.118	-0.127	-0.346	-0.229	-0.113	-0.016	-0.242	0.204	0.044	-0.047	-0.081	0.341
		**p-value**	0.93	0.266	0.711	0.558	0.526	0.077	0.251	0.576	0.937	0.225	0.297	0.826	0.814	0.684	0.076
	**Lobule X**	**rho**	0.269	0.453	0.296	0.162	0.129	0.106	0.389	-0.003	0.14	0.398	-0.144	-0.236	-0.343	**-0.504**	-0.152
		**p-value**	0.174	0.018	0.133	0.419	0.522	0.597	0.045	0.988	0.486	0.04	0.463	0.226	0.074	**0.006**	0.44
	**Dentate nuclei**	**rho**	0.105	0.329	0.085	0.088	-0.108	0.017	0.036	-0.035	-0.053	0.043	0.014	-0.001	-0.25	**-0.521**	-0.061
		**p-value**	0.601	0.094	0.674	0.663	0.592	0.932	0.859	0.861	0.794	0.83	0.943	0.996	0.199	**0.004**	0.759
	**Interposed nuclei**	**rho**	0.231	0.225	0.207	-0.029	-0.239	-0.091	0.045	0.135	-0.122	0.042	-0.147	-0.232	-0.353	-0.249	-0.036
		**p-value**	0.247	0.259	0.301	0.885	0.229	0.651	0.824	0.501	0.544	0.834	0.455	0.235	0.065	0.2	0.854
	**Fastigial nuclei**	**rho**	0.075	-0.081	0.14	0.027	-0.302	0.205	0.122	-0.101	-0.028	0.113	-0.091	-0.111	-0.18	-0.144	-0.143
		**p-value**	0.711	0.69	0.485	0.892	0.125	0.304	0.545	0.616	0.891	0.576	0.645	0.574	0.36	0.464	0.469
		**rho**	0.068	-0.015	0.122	-0.002	0.07	-0.229	-0.102	0.004	-0.158	-0.04	0.188	0.142	-0.101	-0.278	0.322

	**Whole cerebellum**	**p-value**	0.736	0.94	0.545	0.994	0.728	0.25	0.614	0.984	0.432	0.842	0.339	0.47	0.61	0.152	0.095
	**ALS-FTD *C9orf72* (n=12)**		**Memory**	**Everyday skills**	**Self-care skills**	**Mood changes**	**Odd beliefs**	**Abnormal behaviours**	**Eating habits**	**Sleep**	**Stereotypic and motor behaviours**	**Reduced motivation**	**Attention**	**Memory**	**Fluency**	**Language**	**Visuospatial**
**Thalamus**	**AV**	**rho**	0.018	0.486	-0.03	0.522	0.008	0.021	-0.175	0.6	0.064	0.489	0.412	0.091	0.061	-0.091	0.546
		**p-value**	0.957	0.109	0.927	0.082	0.981	0.948	0.587	0.039	0.844	0.107	0.236	0.802	0.867	0.802	0.102
	**LD**	**rho**	-0.11	0.043	-0.198	0.125	0.164	-0.34	-0.135	0.436	0.17	0.151	-0.129	-0.353	0.226	-0.049	-0.067
		**p-value**	0.735	0.895	0.538	0.699	0.61	0.279	0.675	0.157	0.597	0.639	0.722	0.318	0.53	0.894	0.853
	**LP**	**rho**	-0.067	0.479	-0.116	0.439	-0.047	-0.021	-0.25	0.018	-0.106	-0.018	-0.037	0.097	-0.526	0.037	0.055
		**p-value**	0.836	0.115	0.72	0.153	0.885	0.948	0.434	0.956	0.742	0.957	0.919	0.789	0.118	0.92	0.88
	**VA**	**rho**	-0.141	0.243	0.007	0.322	0.106	-0.078	-0.296	0.7	-0.142	0.281	0.277	0.152	0.098	-0.348	0.337
		**p-value**	0.661	0.447	0.982	0.308	0.744	0.81	0.35	0.011	0.66	0.376	0.439	0.675	0.788	0.325	0.34
	**VLa**	**rho**	-0.421	0.221	0.037	0.239	-0.059	-0.128	-0.489	0.207	-0.525	-0.19	0.142	0.438	-0.287	-0.116	-0.031
		**p-value**	0.173	0.489	0.908	0.454	0.856	0.693	0.107	0.518	0.08	0.555	0.696	0.206	0.421	0.75	0.933
	**VLp**	**rho**	-0.378	0.3	-0.052	0.314	-0.086	-0.106	-0.521	0.114	-0.482	-0.162	0.197	0.383	-0.312	-0.061	0.055
		**p-value**	0.226	0.343	0.872	0.32	0.791	0.742	0.083	0.724	0.112	0.616	0.586	0.275	0.38	0.867	0.88
	**VPL**	**rho**	-0.322	0.139	-0.134	0.196	0.063	-0.092	-0.51	-0.325	-0.411	-0.457	0.049	0.237	-0.44	-0.232	-0.129
		**p-value**	0.308	0.666	0.677	0.541	0.847	0.776	0.09	0.303	0.184	0.135	0.893	0.51	0.203	0.519	0.723
	**VM**	**rho**	-0.152	0.15	-0.134	0.246	0.152	0.035	-0.389	-0.393	-0.298	-0.482	-0.037	0.079	-0.41	-0.36	-0.141
		**p-value**	0.637	0.642	0.677	0.44	0.636	0.913	0.212	0.206	0.347	0.113	0.919	0.828	0.24	0.307	0.697
	**Intralaminar**	**rho**	-0.29	0.139	-0.093	0.232	-0.242	0.085	-0.253	-0.104	-0.348	-0.134	0.579	0.347	0.239	0.128	0.264
		**p-value**	0.361	0.666	0.773	0.468	0.448	0.793	0.427	0.749	0.268	0.679	0.08	0.327	0.507	0.724	0.461
	**Midline**	**rho**	0.049	0.089	-0.022	0.236	-0.344	0.355	0.118	0.014	0.05	0.485	**0.794**	0.243	0.294	0.311	0.497
		**p-value**	0.879	0.783	0.945	0.461	0.274	0.258	0.716	0.965	0.878	0.11	**0.006**	0.498	0.41	0.382	0.144
	**MD**	**rho**	-0.315	0.125	-0.299	0.361	-0.195	-0.064	-0.292	-0.004	-0.213	-0.056	0.529	0.213	0.19	0.177	0.141
		**p-value**	0.319	0.699	0.346	0.249	0.543	0.844	0.356	0.991	0.507	0.862	0.116	0.555	0.6	0.625	0.697
	**LGN**	**rho**	-0.18	0.096	-0.112	0.232	0.164	-0.05	-0.303	-0.522	-0.27	-0.587	-0.172	-0.122	-0.379	-0.372	-0.362
		**p-value**	0.575	0.766	0.729	0.468	0.61	0.878	0.338	0.082	0.397	0.045	0.634	0.738	0.28	0.29	0.304
	**MGN**	**rho**	-0.544	0.154	0.019	0.082	-0.086	-0.255	-0.513	-0.339	-0.44	-0.432	0.332	0.553	-0.128	0.171	-0.117

		**p-value**	0.067	0.634	0.954	0.8	0.791	0.423	0.088	0.281	0.153	0.16	0.348	0.097	0.724	0.637	0.748
	**Pulvinar**	**rho**	-0.389	0.186	-0.011	0.293	0.031	-0.128	-0.521	-0.15	-0.504	-0.355	0.197	0.201	-0.171	-0.25	-0.129
		**p-value**	0.212	0.563	0.972	0.355	0.923	0.693	0.083	0.642	0.095	0.257	0.586	0.578	0.636	0.486	0.723
	**Whole thalamus**	**rho**	-0.375	0.246	-0.067	0.393	-0.051	-0.035	-0.496	-0.082	-0.447	-0.214	0.339	0.328	-0.165	-0.091	0.018
		**p-value**	0.23	0.44	0.836	0.206	0.875	0.913	0.101	0.8	0.145	0.503	0.339	0.354	0.648	0.802	0.96
**Cerebellum**	**Lobule I-IV**	**rho**	-0.276	-0.514	-0.034	-0.575	-0.121	-0.156	0.082	-0.339	-0.163	-0.496	-0.326	0.28	-0.061	0.372	-0.522
		**p-value**	0.386	0.087	0.917	0.05	0.708	0.628	0.8	0.281	0.612	0.101	0.358	0.434	0.867	0.29	0.122
	**Lobule V**	**rho**	-0.307	-0.046	-0.03	-0.086	-0.027	0.007	-0.3	-0.457	-0.348	-0.527	0.197	0.602	-0.147	0.116	-0.08
		**p-value**	0.331	0.886	0.927	0.791	0.933	0.983	0.344	0.135	0.268	0.078	0.586	0.066	0.686	0.75	0.827
	**Lobule VI**	**rho**	0.297	0.307	0.116	0.422	0.289	0.575	-0.029	-0.397	0.092	-0.102	0.449	0.359	-0.055	-0.049	0.215
		**p-value**	0.349	0.331	0.72	0.172	0.362	0.051	0.93	0.202	0.776	0.753	0.193	0.309	0.88	0.894	0.551
	**Lobule VIIa-CrusI**	**rho**	0.074	0.139	0.284	0.189	-0.113	0.44	0.26	-0.389	-0.014	-0.067	0.677	0.359	0.502	0.177	0.288
		**p-value**	0.819	0.666	0.372	0.556	0.726	0.153	0.414	0.211	0.965	0.837	0.032	0.309	0.14	0.625	0.419
	**Lobule VIIa-CrusII**	**rho**	-0.269	-0.082	0.414	-0.079	0.191	-0.163	0.05	0.046	-0.163	-0.288	-0.129	-0.061	0.459	-0.335	-0.362
		**p-value**	0.399	0.8	0.181	0.808	0.551	0.612	0.878	0.886	0.612	0.364	0.722	0.868	0.182	0.343	0.304
	**Lobule VIIb**	**rho**	-0.064	0.071	0.455	-0.004	0.367	0.121	0.032	-0.175	-0.064	-0.369	0.037	0.255	0.428	-0.482	-0.104
		**p-value**	0.844	0.825	0.137	0.991	0.24	0.709	0.921	0.586	0.844	0.238	0.919	0.476	0.217	0.159	0.774
	**Lobule VIIIa**	**rho**	0.057	0.079	0.321	0.029	0.383	0.156	0.061	-0.264	-0.05	-0.478	-0.332	0.043	0.043	-0.598	-0.264
		**p-value**	0.861	0.808	0.309	0.93	0.219	0.628	0.852	0.406	0.878	0.116	0.348	0.907	0.907	0.068	0.461
	**Lobule VIIIb**	**rho**	0.049	-0.046	0.175	0.004	0.371	0.177	0.05	-0.204	-0.057	-0.489	-0.271	0.061	-0.018	-0.573	-0.202
		**p-value**	0.879	0.886	0.586	0.991	0.235	0.581	0.878	0.526	0.861	0.107	0.449	0.868	0.96	0.083	0.575
	**Lobule IX**	**rho**	0.205	0.35	0.153	0.418	0.426	0.319	-0.168	-0.343	0.014	-0.179	-0.068	-0.055	-0.593	-0.591	0.031
		**p-value**	0.523	0.265	0.635	0.176	0.167	0.312	0.603	0.275	0.965	0.577	0.853	0.881	0.071	0.072	0.933
	**Lobule X**	**rho**	-0.226	-0.271	-0.019	-0.107	0.219	-0.099	-0.014	-0.622	-0.078	**-0.717**	-0.197	-0.14	0.343	-0.201	-0.583
		**p-value**	0.48	0.393	0.954	0.74	0.494	0.759	0.965	0.031	0.81	**0.009**	0.586	0.7	0.333	0.577	0.077
	**Dentate nuclei**	**rho**	0.24	0.068	0.239	-0.032	-0.387	0.277	0.56	0.057	0.184	0.397	0.4	-0.043	0.593	0.183	0.472
		**p-value**	0.452	0.834	0.455	0.921	0.214	0.384	0.058	0.86	0.566	0.201	0.252	0.907	0.071	0.613	0.168
		**rho**	0.29	0.139	0.269	-0.025	-0.391	0.362	0.342	0.271	-0.035	0.478	0.277	0.213	0.037	0.159	0.399

	**Interposed nuclei**	**p-value**	0.361	0.666	0.398	0.939	0.209	0.248	0.276	0.393	0.913	0.116	0.439	0.555	0.92	0.662	0.254
	**Fastigial nuclei**	**rho**	-0.014	-0.132	0.328	-0.361	-0.402	0.057	0.382	-0.036	-0.17	0.004	-0.351	0.109	-0.153	0.28	-0.276
		**p-value**	0.965	0.682	0.297	0.249	0.195	0.861	0.221	0.912	0.597	0.991	0.32	0.763	0.673	0.432	0.44
	**Whole cerebellum**	**rho**	-0.131	-0.036	0.332	-0.029	0.168	0.142	0.046	-0.347	-0.192	-0.464	0.049	0.267	0.3	-0.293	-0.239
		**p-value**	0.685	0.912	0.291	0.93	0.602	0.66	0.886	0.27	0.551	0.129	0.893	0.455	0.4	0.412	0.506
	**ALS sporadic (n=49)**		**Memory**	**Everyday skills**	**Self-care skills**	**Mood changes**	**Odd beliefs**	**Abnormal behaviours**	**Eating habits**	**Sleep**	**Stereotypic and motor behaviours**	**Reduced motivation**	**Attention**	**Memory**	**Fluency**	**Language**	**Visuospatial**
**Thalamus**	**AV**	**rho**	0.251	-0.024	0.009	0.087	0.245	0.082	0.244	0.112	0.165	0.084	0.073	0.174	-0.123	0.194	-0.112
		**p-value**	0.081	0.874	0.954	0.553	0.09	0.576	0.095	0.454	0.263	0.565	0.68	0.324	0.488	0.271	0.529
	**LD**	**rho**	-0.119	-0.14	-0.071	-0.194	-0.112	-0.062	-0.265	0.085	-0.248	-0.323	-0.087	0.238	0.087	0.341	-0.328
		**p-value**	0.416	0.348	0.631	0.182	0.442	0.67	0.069	0.57	0.089	0.024	0.625	0.176	0.626	0.048	0.058
	**LP**	**rho**	-0.051	0.034	0.091	-0.127	0.092	-0.136	-0.146	0.102	-0.164	-0.269	0.008	-0.073	0.015	0.276	-0.269
		**p-value**	0.729	0.822	0.538	0.383	0.53	0.35	0.321	0.493	0.264	0.062	0.964	0.682	0.931	0.115	0.123
	**VA**	**rho**	0.143	0.054	0.01	0.044	0.112	0.125	0.14	-0.165	0.111	0.151	0.167	-0.117	-0.059	-0.185	0.028
		**p-value**	0.328	0.716	0.948	0.762	0.442	0.393	0.343	0.268	0.452	0.302	0.346	0.511	0.742	0.296	0.874
	**VLa**	**rho**	0.119	0.095	0.024	0.021	0.102	0.078	0.234	-0.114	0.076	0.316	0.125	-0.259	-0.085	-0.295	0.241
		**p-value**	0.416	0.524	0.869	0.887	0.485	0.596	0.11	0.445	0.606	0.027	0.482	0.139	0.633	0.09	0.17
	**VLp**	**rho**	0.197	0.165	0.141	0.077	0.061	0.157	0.243	-0.029	0.12	**0.367**	0.176	-0.314	-0.032	-0.167	0.342
		**p-value**	0.174	0.268	0.341	0.601	0.676	0.282	0.097	0.849	0.417	**0.009**	0.319	0.07	0.859	0.346	0.048
	**VPL**	**rho**	0.187	0.103	0.17	0.07	-0.01	0.118	0.113	-0.034	0.143	0.296	0.303	-0.178	-0.105	-0.244	0.353
		**p-value**	0.198	0.492	0.249	0.633	0.945	0.418	0.445	0.822	0.331	0.039	0.081	0.314	0.553	0.165	0.04
	**VM**	**rho**	0.151	0.055	0.121	0.046	-0.01	0.027	-0.006	-0.107	0.095	0.191	0.337	-0.064	-0.094	-0.135	0.279
		**p-value**	0.3	0.714	0.413	0.753	0.945	0.854	0.967	0.472	0.522	0.188	0.051	0.72	0.595	0.445	0.109
	**Intralaminar**	**rho**	0.282	0.073	0.224	0.089	0.092	0.11	-0.014	0.021	0.093	0.062	0.399	-0.004	-0.118	0.064	0.049
		**p-value**	0.05	0.628	0.126	0.544	0.53	0.451	0.926	0.887	0.529	0.674	0.019	0.981	0.505	0.721	0.784
	**Midline**	**rho**	0.309	0.084	0.243	0.11	0.133	0.144	-0.018	0.058	0.018	0	0.206	-0.038	-0.086	0.024	-0.141
		**p-value**	0.031	0.574	0.097	0.45	0.363	0.324	0.903	0.701	0.904	0.998	0.242	0.833	0.629	0.894	0.426
	**MD**	**rho**	-0.053	0.031	0.039	-0.13	0.071	-0.09	-0.136	0.057	-0.116	-0.107	-0.007	-0.135	-0.105	0.085	-0.291

		**p-value**	0.716	0.834	0.794	0.372	0.626	0.54	0.357	0.703	0.43	0.466	0.967	0.447	0.554	0.633	0.095
	**LGN**	**rho**	-0.069	-0.166	0.02	-0.214	-0.245	-0.258	-0.259	-0.218	-0.24	-0.342	0.405	0.169	-0.012	0.087	0.183
		**p-value**	0.639	0.266	0.892	0.139	0.09	0.073	0.076	0.141	0.101	0.016	0.018	0.34	0.945	0.624	0.299
	**MGN**	**rho**	0.159	0.08	0.113	-0.004	-0.082	-0.068	0.098	-0.035	-0.021	0.085	0.388	-0.028	-0.421	-0.077	0.258
		**p-value**	0.276	0.593	0.443	0.979	0.577	0.642	0.508	0.818	0.888	0.564	0.024	0.873	0.013	0.667	0.141
	**Pulvinar**	**rho**	-0.059	-0.023	0.157	-0.125	0.01	-0.105	-0.176	-0.019	-0.081	-0.22	0.184	0.249	0.062	0.073	0.148
		**p-value**	0.687	0.878	0.286	0.392	0.945	0.473	0.232	0.897	0.582	0.129	0.297	0.156	0.729	0.68	0.405
	**Whole thalamus**	**rho**	0.142	0.085	0.184	-0.012	0.041	0.017	0.002	-0.024	0.058	0.074	0.337	-0.015	-0.092	-0.006	0.159
		**p-value**	0.33	0.568	0.211	0.937	0.781	0.907	0.989	0.873	0.694	0.611	0.051	0.934	0.607	0.972	0.371
**Cerebellum**	**Lobule I-IV**	**rho**	0.169	0.348	0.341	0.108	-0.01	0.211	0.287	0.241	0.274	0.364	-0.268	-0.245	0.026	0.064	0.103
		**p-value**	0.247	0.016	0.018	0.459	0.945	0.145	0.048	0.102	0.06	0.01	0.125	0.163	0.884	0.718	0.563
	**Lobule V**	**rho**	0.249	0.29	0.262	0.24	0.133	0.044	**0.369**	0.21	0.287	0.338	-0.024	-0.074	-0.262	0.174	-0.03
		**p-value**	0.084	0.048	0.072	0.097	0.363	0.766	**0.01**	0.156	0.048	0.018	0.893	0.677	0.134	0.325	0.865
	**Lobule VI**	**rho**	0.187	**0.405**	0.337	0.204	0.071	0.099	0.056	0.262	0.089	0.309	-0.138	-0.031	0.158	0.227	-0.011
		**p-value**	0.199	**0.005**	0.019	0.159	0.626	0.497	0.705	0.075	0.548	0.03	0.437	0.861	0.373	0.196	0.952
	**Lobule VIIa-CrusI**	**rho**	0.171	0.166	0	0.125	0	-0.018	0.174	0.095	0.075	0.272	0.179	0.011	-0.231	0.165	0.109
		**p-value**	0.239	0.264	0.998	0.391	1	0.903	0.237	0.525	0.614	0.058	0.312	0.95	0.189	0.351	0.54
	**Lobule VIIa-CrusII**	**rho**	0.091	0.167	0.094	0.054	0.194	0.115	0.187	0.027	0.273	0.329	0.063	-0.052	-0.315	0.043	0.181
		**p-value**	0.534	0.261	0.525	0.715	0.182	0.429	0.204	0.856	0.06	0.021	0.723	0.771	0.07	0.811	0.304
	**Lobule VIIb**	**rho**	0.059	0.326	0.253	0.193	0.102	0.135	0.163	0.225	0.358	0.275	-0.173	-0.047	-0.125	0.139	0.119
		**p-value**	0.685	0.025	0.083	0.184	0.485	0.356	0.269	0.129	0.013	0.056	0.328	0.793	0.482	0.434	0.503
	**Lobule VIIIa**	**rho**	0.146	0.148	0.197	0.164	0.041	0.143	0.078	0.246	0.367	0.142	-0.244	-0.021	-0.047	0.311	-0.051
		**p-value**	0.317	0.322	0.18	0.261	0.781	0.328	0.6	0.096	0.01	0.33	0.164	0.908	0.791	0.073	0.773
	**Lobule VIIIb**	**rho**	-0.047	-0.059	0.044	-0.083	-0.041	-0.079	-0.102	0.003	0.096	-0.172	-0.212	0.206	-0.036	0.344	-0.084
		**p-value**	0.747	0.691	0.768	0.573	0.781	0.589	0.49	0.982	0.517	0.237	0.228	0.244	0.84	0.047	0.638
	**Lobule IX**	**rho**	0.061	0.144	0.087	0.025	0	-0.076	0.122	0.044	0.138	0.015	-0.14	-0.034	-0.266	0.327	0.159
		**p-value**	0.677	0.335	0.557	0.863	1	0.605	0.41	0.767	0.349	0.92	0.429	0.848	0.128	0.059	0.368
	**Lobule X**	**rho**	0.028	0.031	0.065	0.066	0.174	-0.032	0.131	0.134	0.257	0.141	-0.18	0	-0.076	0.162	-0.213

		**p-value**	0.847	0.834	0.663	0.654	0.233	0.826	0.376	0.369	0.077	0.334	0.308	0.999	0.668	0.359	0.228
	**Dentate nuclei**	**rho**	0.259	**0.38**	0.3	0.3	0.153	0.182	0.121	0.126	0.295	0.206	0.071	0.139	-0.172	0.245	0.082
		**p-value**	0.072	**0.008**	0.038	0.036	0.294	0.212	0.411	0.399	0.042	0.155	0.688	0.432	0.33	0.163	0.646
	**Interposed nuclei**	**rho**	0.216	0.097	0.058	0.078	-0.071	0.218	0.088	0.091	0.213	0.242	0.159	-0.011	-0.03	-0.014	-0.139
		**p-value**	0.135	0.516	0.697	0.596	0.626	0.131	0.551	0.544	0.146	0.093	0.368	0.95	0.867	0.936	0.432
	**Fastigial nuclei**	**rho**	0.038	-0.067	-0.168	-0.043	0.092	0.23	0.25	0.052	0.272	0.274	-0.091	-0.246	-0.274	-0.309	-0.15
		**p-value**	0.795	0.654	0.253	0.769	0.53	0.112	0.087	0.73	0.062	0.056	0.607	0.161	0.117	0.075	0.398
	**Whole cerebellum**	**rho**	0.185	0.292	0.184	0.212	0.143	0.117	0.273	0.24	0.332	0.36	-0.163	0.003	-0.157	0.229	0.018
		**p-value**	0.203	0.046	0.21	0.144	0.327	0.422	0.061	0.105	0.021	0.011	0.358	0.985	0.375	0.194	0.922
